# DNA Topoisomerase II Is Involved in Regulation of Cyst Wall Protein Genes and Differentiation in *Giardia lamblia*

**DOI:** 10.1371/journal.pntd.0002218

**Published:** 2013-05-16

**Authors:** Bo-Chi Lin, Li-Hsin Su, Shih-Che Weng, Yu-Jiao Pan, Nei-Li Chan, Tsai-Kun Li, Hsin-Chih Wang, Chin-Hung Sun

**Affiliations:** 1 Department of Parasitology, College of Medicine, National Taiwan University, Taipei, Taiwan, Republic of China; 2 Institute of Biochemistry and Molecular Biology, College of Medicine, National Taiwan University, Taipei, Taiwan, Republic of China; 3 Department and Graduate Institute of Microbiology, College of Medicine, National Taiwan University, Taipei, Taiwan, Republic of China; Georgetown University, United States of America

## Abstract

The protozoan *Giardia lamblia* differentiates into infectious cysts within the human intestinal tract for disease transmission. Expression of the cyst wall protein (*cwp*) genes increases with similar kinetics during encystation. However, little is known how their gene regulation shares common mechanisms. DNA topoisomerases maintain normal topology of genomic DNA. They are necessary for cell proliferation and tissue development as they are involved in transcription, DNA replication, and chromosome condensation. A putative topoisomerase II (*topo II*) gene has been identified in the *G. lamblia* genome. We asked whether Topo II could regulate *Giardia* encystation. We found that Topo II was present in cell nuclei and its gene was up-regulated during encystation. Topo II has typical ATPase and DNA cleavage activity of type II topoisomerases. Mutation analysis revealed that the catalytic important Tyr residue and cleavage domain are important for Topo II function. We used etoposide-mediated topoisomerase immunoprecipitation assays to confirm the binding of Topo II to the *cwp* promoters *in vivo*. Interestingly, Topo II overexpression increased the levels of *cwp* gene expression and cyst formation. Microarray analysis identified up-regulation of *cwp* and specific *vsp* genes by Topo II. We also found that the type II topoisomerase inhibitor etoposide has growth inhibition effect on *Giardia*. Addition of etoposide significantly decreased the levels of *cwp* gene expression and cyst formation. Our results suggest that Topo II has been functionally conserved during evolution and that Topo II plays important roles in induction of the *cwp* genes, which is key to *Giardia* differentiation into cysts.

## Author Summary

*Giardia lamblia* becomes infective by differentiation into water-resistant cysts. During encystation, cyst wall proteins (CWPs) are highly synthesized and are targeted to the cyst wall. However, little is known about the regulation mechanisms of these genes. DNA topoisomerases can resolve the topological problems and are needed for a variety of key cellular functions, including cell proliferation, cell differentiation and organ development in higher eukaryotes. We found that giardial Topo II was highly expressed during encystation. Topo II is present in *Giardia* nuclei and is associated with the encystation-induced *cwp* gene promoters. Topo II has typical DNA cleavage activity of type II topoisomerases. Interestingly, overexpression of Topo II can induce *cwp* gene expression and cyst formation. Addition of a type II topoisomerase inhibitor, etoposide, significantly decreased the levels of *cwp* gene expression and cyst formation. Etoposide also has growth inhibition effect on *Giardia*. Our results suggest that Topo II plays an important role in induction of encystation by up-regulation of the *cwp* gene expression. Our results provide insights into the function of Topo II in parasite differentiation into cysts and help develop ways to interrupt the parasite life cycle.

## Introduction

*Giardia lamblia* is an intestinal protozoan parasite causing outbreaks of infectious diarrheal diseases worldwide with an estimated 280 million cases of giardiasis yearly [1], [2], [3]. It has been isolated from various other animals that may act as reservoirs for human infection [4]. Giardiasis is prevalent in developing countries of the tropics and also in travelers to developed countries [4]. It contributes greatly to malnutrition and malabsorption leading to childhood mortality [5]. Patients with giardiasis may have a post-infectious irritable bowel syndrome [6], [7]. Transmission of giardiasis occurs through ingestion of infective cysts from contaminated water or food [4]. After passage through the stomach, the trophozoites emerge in the small intestine and colonize the human small intestine. They may differentiate into infective cysts when carried downstream to lower intestine [1], [4].

*G. lamblia* is a valuable model for other intestinal protozoan parasites as its life cycle can be reproduced *in vitro* [1], [4]. *G. lamblia* also raises great biological interest for understanding the progress of eukaryotic evolution [8]. *G. lamblia* has fewer components of DNA synthesis, transcription, and RNA processing [1], [9]. However, massive gene expansion happened in the Nek kinase protein family [10], possibly due to the requirement of the Nek kinases for flagellar function and cell motility. Many aspects of giardial transcription are unusual. Very short 5′-flanking region (<65 bp) with no consensus TATA boxes or typical *cis*-acting elements are sufficient for expression of many genes [11], [12], [13], [14]. Interestingly, AT-rich sequences spanning the transcription start sites of many genes are essential for promoter activity and play a predominant role in determining the positions of the transcription start sites, functionally similar to the initiator elements in higher eukaryotes [11], [12], [13], [15].

*G. lamblia* is transmitted by differentiation into infective cysts, which are protectively walled and can survive in water [1], [4]. Key components of cyst wall include some proteins and polysaccharides [16], [17], [18], [19], [20], [21]. Three known cyst wall proteins (CWPs) are highly synthesized in a concerted manner during differentiation into cysts [17], [18], [19], suggesting the importance of gene regulation. Several transcription factors regulating *cwp* gene expression have been identified [15], [22], [23], [24], [25], [26], [27], [28]. Expression of variant surface proteins may be regulated by a microRNA mediated post transcriptional regulation system [29], but little is known of relative regulation in the CWP expression. *Giardia* encystation has been proposed to link to cell cycle regulation and Cdk2 pathway may be involved in activation of Myb2 and up-regulation of *cwp* genes [30], [31], [32].

Topoisomerases can resolve the topological problems of chromosomes in both prokaryotes and eukaryotes [33], [34]. They are necessary for cell growth, tissue development, or cell cycle progression as they are involved in DNA replication, transcription, recombination, chromosome condensation, and segregation of newly replicated chromosomes [33], [34]. Type I topoisomerases work by cleaving one strand of DNA, but Type II topoisomerases work by cleaving two strands of DNA [33], [34], [35]. Two isoforms of type II topoisomerases have been found in mammals, IIα and IIβ [36]. Topoisomerase IIα plays an essential role and is expressed at a higher level during cell growth and proliferation [36], [37]. Topoisomerase IIβ has an essential role in neuronal development by inducing transcription of specific genes required for neuronal development [38], [39]. Location of its target genes is closed to AT-rich intergenic regions [38], [39]. Topoisomerase II enhances transcription by binding to nucleosome-free promoters and recruiting RNA polymerase II in yeast [40].

Type II topoisomerases create a transient double stranded DNA break by transesterification of a specific Tyr of the enzyme cleavage domain and a phosphodiester bond of DNA [33], [34]. They further act by passing a second duplex through the DNA break. The ATPase domain has ATP hydrolysis activity to provide energy for movement of DNA along enzyme [41]. An interdomain between ATPase and cleavage domain (amino acids 357–407) of human topoisomerase IIα has been identified to be important for interdomain communication [42]. The C terminal regions of topoisomerases II are species specific and may help design therapeutic drugs [43], [44], [45], [46].

DNA topoisomerases are potential therapeutic targets for drug discovery. Many antitumor agents act through inhibiting topoisomerase activity in caner cells [47]. Many anti-bacterial and anti-Apicomplex parasite drugs act by inhibiting DNA topoisomerases [48], [49]. Mammalian type II topoisomerase inhibitors, such as etoposide (also known as VP-16) and doxorubicin, target topoisomerases IIα and β [50]. Etoposide traps the cleavage complex to prevent religation of DNA, resulting in double stranded DNA break and cell apoptosis [51], [52]. Drug resistance is correlated with mutation of topoisomerases, reduced topoisomerase II activity or decreased amount of enzyme [53], [54]. Metronidazole has been used often in the treatment of *Giardia* infection, but resistance and side effect limits its use [55]. Studies of topoisomerases will provide therapeutic perspectives in *Giardia* and other important intestinal protozoan pathogens.

A putative topoisomerase II (Topo II) has been identified in *G. lamblia* genome [56]. During encystation, a trophozoite may differentiate into a cyst by dividing 2 nuclei and by replicating DNA, generating a cyst with 4 nuclei [1]. It has been shown that homologous recombination may occur in *Giardia* cyst nuclei [57]. Because type II topoisomerases play critical roles in chromosome replication, cell cycle and tissue development in many eukaryotes, we asked whether Topo II could be important for *Giardia* differentiation into dormant cysts. We found that the expression levels of the *Giardia* Topo II increased during encystation. In addition, Topo II has typical ATPase, DNA binding, and DNA cleavage activity of type II topoisomerases. We also found that the levels of cyst formation and the *cwp1-3* and *myb2* gene expression increased by Topo II overexpression, suggesting that Topo II may be an important factor involved in activation of these gene expression and *Giardia* encystation. We used a method similar to chromatin immunoprecipitation (ChIP) assays, etoposide-mediated topoisomerase immunoprecipitation assays [38] to confirm the binding of Topo II to these gene promoters *in vivo*. We also tested the effect of a type II topoisomerase inhibitor, etoposide, and found that it inhibited *Giardia* growth and decreased the levels of cyst formation and the *cwp1-3* and *myb2* gene expression. Because etoposide has side effect [47], further studies are required to find more suitable topoisomerase inhibitors to inhibit *Giardia* growth but has less side effect. Our results provide insights into the role of type II topoisomerase in inducing *Giardia* differentiation into dormant cysts and into the development of better drugs for treatment of giardiasis.

## Materials and Methods

### *G. lamblia* Culture

Trophozoites of *G. lamblia* WB (ATCC 50803), clone C6, were cultured in modified TYI-S33 medium [58]. Encystation was performed as previously described [19]. Briefly, trophozoites that were grown to late log phase in growth medium were harvested and encysted for 24 h in TYI-S-33 medium containing 12.5 mg/ml bovine bile at pH 7.8 at a beginning density of 5×10^5^ cells/ml.

### Cyst Count

Cyst count was performed on the stationary phase cultures (∼2×10^6^ cells/ml) during vegetative growth as previously described [59]. Cells were subcultured in growth medium with suitable selection drugs at an initial density of 1×10^6^ cells/ml. Cells seeded at this density became confluent within 24 h. Confluent cultures were maintained for an additional 8 h to ensure that the cultures were in stationary phase (at a density of ∼2×10^6^ cells/ml). Cyst count was performed on these stationary phase cultures. Cultures were chilled and cells were washed twice in double-distilled water at 4°C and trophozoites were lysed by incubation in double-distilled water overnight at 4°C. Cysts were washed three times in double-distilled water at 4°C. Water-resistant cysts were counted in a hemacytometer chamber. Cyst count was also performed on 24 h encysting cultures.

### Isolation and Analysis of the *Topo II* Gene

The *G. lamblia* genome database (http://www.giardiadb.org/giardiadb/) [9], [60] was searched with the amino acid sequence of the human topoisomerase IIα (GenBank accession number **NP_001058.2**) using the BLAST program [61]. This search detected one putative homologue for topoisomerase II (Topo II) that has been reported previously (GenBank accession number **XP_001708897.1**, open reading frame 16975 in the *G. lamblia* genome database). The Topo II coding region with 221 bp of 5′- flanking region was cloned and the nucleotide sequence was determined. The *topo II* gene sequence in the database was correct. To isolate the cDNA of the *topo II* gene, we performed RT-PCR with *topo II*-specific primers using total RNA from *G. lamblia*. For RT-PCR, 5 µg of DNase-treated total RNA from vegetative and 24 h encysting cells was mixed with oligo (dT)12–18 and random hexamers and Superscript II RNase H- reverse transcriptase (Invitrogen). Synthesized cDNA was used as a template in subsequent PCR with primers topo IIF and topo IIR. Oligonucleotides used in this study are listed in Table S1. Genomic and RT-PCR products were cloned into pGEM-T easy vector (Promega) and sequenced (Applied Biosystems, ABI).

### RNA Extraction, RT-PCR and Quantitative Real-Time PCR Analysis

Total RNA was extracted from *G. lamblia* cell line at the differentiation stages indicated in figure legends using TRIzol reagent (Invitrogen). For RT-PCR, 5 µg of DNase-treated total RNA was mixed with oligo (dT)12–18 and random hexamers and Superscript II RNase H^−^ reverse transcriptase (Invitrogen). Synthesized cDNA was used as a template in subsequent PCR. Semi-quantitative RT-PCR analysis of *topo II* (**XP_001708897.1**, open reading frame 16975), *topo II-ha*, *cwp1* (**U09330**, open reading frame 5638), *cwp2* (**U28965**, open reading frame 5435), *cwp3* (**AY061927**, open reading frame 2421), *myb2* (**AY082882**, open reading frame 8722), *ran* (**U02589**, open reading frame 15869), and 18 S ribosomal RNA (**M54878**, open reading frame r0019) gene expression was performed using primers topo II828F and topo II1311R, topo IIHAF and HAR, cwp1F and cwp1R, cwp2F and cwp2R, cwp3F and cwp3R, myb2F and myb2R, ranF and ranR, 18SrealF and 18SrealR, respectively. For quantitative real-time PCR, SYBR Green PCR master mixture was used (Kapa Biosystems). PCR was performed using an Applied Biosystems PRISMTM 7900 Sequence Detection System (Applied Biosystems). Specific primers were designed for detection of the *topo II*, *topo II-ha*, *cwp1*, *cwp2*, *cwp3*, *myb2*, *ran*, and 18 S ribosomal RNA genes: topo IIrealF and topo IIrealR; topo IIHAF and HAR; cwp1realF and cwp1realR; cwp2realF and cwp2realR; cwp3realF and cwp3realR; myb2realF and myb2realR; ranrealF and ranrealR; 18SrealF and 18SrealR. Two independently generated stably transfected lines were made from each construct and each of these cell lines was assayed three separate times. The results are expressed as relative expression level over control. Student's *t*-tests were used to determine statistical significance of differences between samples.

### Plasmid Construction

All constructs were verified by DNA sequencing with a BigDye Terminator 3.1 DNA Sequencing kit and an Applied Biosystems 3100 DNA Analyser (Applied Biosystems). Plasmid 5′Δ5N-Pac was a gift from Dr. Steven Singer and Dr. Theodore Nash [62]. To make construct pPTopo II, the *topo II* gene and its 221 bp of 5′- flanking region were amplified with oligonucleotides topo IIKF and topo IIAR, digested with KpnI and AvrII, and cloned into KpnI and XbaI digested pPop2N [63]. To make construct pPTopo IIm1, the *topo II* gene was amplified using two primer pairs topo IIm1F and topo IIAR, and topo IIm1R and topo IIKF. The two PCR products were purified and used as templates for a second PCR. The second PCR also included primers topo IIKF and topo IIAR, and the product was digested with KpnI and AvrII and cloned into the KpnI and XbaI digested pPop2N. To make construct pPTopo IIm2 or pPTopo IIm3, the *topo II* gene was amplified using primers topo IIKF and topo IIm2AR or topo IIm3AR, digested with KpnI and AvrII, and cloned into KpnI and XbaI digested pPop2N [63]. To make construct pPTopo II5, the 300-bp 5′-flanking region of the *topo II* gene was amplified with primers topo II5XF and topo II5NR, digested with XbaI and NcoI, and ligated in place of the NheI/NcoI-excised *ran* promoter sequence in pPop2N. To make construct pPTopo II5m, the 300-bp 5′-flanking region of the *topo II* gene was amplified with primers topo II5XF and topo II5mNR, digested with XbaI and NcoI, and ligated in place of the NheI/NcoI-excised *ran* promoter sequence in pPop2N.

### Transfection, Luciferase Assay, and Western Blot Analysis

Cells transfected with the pP series plasmids containing the *pac* gene were selected and maintained with 54 µg/ml of puromycin as described [62], [64]. The luciferase activity was determined as described [16]. After stable transfection with specific constructs, luciferase activity was determined in vegetative cells at late log/stationary phase (1.5×10^6^ cells/ml) or 24 h encysting cells as described [16] and was measured with an Optocomp I luminometer (MGM Instruments). Two independently generated stably transfected lines were made from each construct and each of these lines was assayed three separate times. Western blots were probed with anti-V5-horseradish peroxidase (Invitrogen), anti-HA monoclonal antibody (1/5000 in blocking buffer; Sigma), anti-CWP1 (1/10000 in blocking buffer) [24], anti-Myb2 (1/5000 in blocking buffer) [27], anti-RAN (1/10000 in blocking buffer) [28], anti-Topo II (1/10000 in blocking buffer) (see below), or preimmune serum (1/5000 in blocking buffer), and detected with peroxidase-conjugated goat anti-mouse IgG (1/5000; Pierce) or peroxidase-conjugated goat anti-rabbit IgG (1/5000; Pierce) and enhanced chemiluminescence (GE Healthcare). Scanned images were analyzed by the ImageJ software (NIH, USA).

### Expression and Purification of Recombinant Topo II Protein

The genomic *topo II* gene was amplified using oligonucleotides topo IIF and topo IIR. The product was cloned into the expression vector pET101/D-TOPO (Invitrogen) in frame with the C-terminal His and V5 tag to generate plasmid pTopo II. To make pTopo IIN expression vector, the *topo II* gene was amplified using primers topo IIF and topo IINR and cloned into the expression vector. To make pTopo IIC expression vector, the *topo II* gene was amplified using primers topo IICF and topo IIR and cloned into the expression vector. To make the pTopo IICm1, pTopo IICm2, or pTopo IICm3 expression vector, the *topo II* gene was amplified using primers topo IICF and topo IIR and specific template, including pPTopo IIm1, pPTopo IIm2, or pPTopo IIm3, and cloned into the expression vector. The pTopo IIN, pTopo IIC, pTopo IICm1, pTopo IICm2, or pTopo IICm3 plasmid was freshly transformed into *Escherichia coli* BL21 Star (DE3) (Invitrogen). An overnight pre-culture was used to start a 250-ml culture. *E. coli* cells were grown to an A600 of 0.5, and then induced with 1 mM isopropyl-D-thiogalactopyranoside (IPTG) (Promega) for 4 h. Bacteria were harvested by centrifugation and sonicated in 10 ml of buffer A (50 mM sodium phosphate, pH 8.0, 300 mM NaCl) containing 10 mM imidazole and protease inhibitor mixture (Sigma). The samples were centrifuged, and the supernatant was mixed with 1 ml of 50% slurry of nickel-nitrilotriacetic acid Superflow (Qiagen). The resin was washed with buffer A containing 20 mM imidazole and eluted with buffer A containing 250 mM imidazole. Fractions containing Topo II, Topo IIN, Topo IIC, Topo IICm1, Topo IICm2, or Topo IICm3 were pooled, dialyzed in 25 mM HEPES pH 7.9, 20 mM KCl, and 15% glycerol, and stored at −70°C. Protein purity and concentration were estimated by Coomassie Blue and silver staining compared with serum albumin. Topo II, Topo IIN, Topo IIC, Topo IICm1, Topo IICm2, or Topo IICm3 was purified to apparent homogeneity (>95%).

### Generation of Anti-Topo II Antibody

Purified Topo II protein was used to generate rabbit polyclonal antibodies through a commercial vendor (Angene, Taipei, Taiwan).

### Immunofluorescence Assay

The pPTopo II, pPTopo IIm1, pPTopo IIm2, or pPTopo IIm3 stable transfectants were cultured in growth medium under puromycin selection. Cells cultured in growth medium or encystation medium for 24 h were harvested, washed in phosphate-buffered saline (PBS), and attached to glass coverslips (2×10^6^ cells/coverslip) and then fixed and stained [16]. Cells were reacted with anti-HA monoclonal antibody (1/300 in blocking buffer; Molecular Probes) and anti-mouse ALEXA 488 (1/500 in blocking buffer, Molecular Probes) as the detector. ProLong antifade kit with 4′,6-diamidino-2-phenylindole (Invitrogen) was used for mounting. Topo II, Topo IIm1, Topo IIm2, or Topo IIm3 was visualized using a Leica TCS SP5 spectral confocal system.

### Electrophoretic Mobility Shift Assay

Double-stranded oligonucleotides specified throughout were 5′-end-labeled as described [11]. Binding reaction mixtures contained the components described [15]. Labeled probe (0.02 pmol) was incubated for 15 min at room temperature with 5 ng of purified Topo II, Topo IIC, Topo IICm1, Topo IICm2, or Topo IICm3 protein in a 20 µl volume supplemented with 0.5 µg of poly (dI-dC) (Sigma). Competition reactions contained 200-fold molar excess of cold oligonucleotides. In an antibody supershift assay, 0.8 µg of an anti-V5-horseradish peroxidase antibody (Bethyl Laboratories) was added to the binding reaction mixture. The mixture was separated on a 6% acrylamide gel by electrophoresis.

### ATPase Assays

ATPase assays were performed using purified Topo II and pyruvate kinase/lactate dehydrogenase reaction as described [43]. Reaction was performed in a 0.2 ml mixture containing 0.4 mM NADH, 2 mM phosphoenolpyruvate, 3 mM ATP, and 8 ng purified Topo II. Some reactions contained 300 ng DNA. Reaction was initiated by mixing with 1 unit of pyruvate kinase and 1.5 units of lactate dehydrogenase and incubating at 37°C. To determine the decrease of NADH concentration, absorbance at A340 nm was measured every 50 seconds for 15 min using a PARADIGM spectrophotometer (Beckman-Coulter). Triplicate samples were monitored. Rate of ATPase activity (V) was calculated as: V = –(ΔO.D.340/Δtime)/cuvette pathlength in cm/6.22 mM^−1^ (6.22 = Millimolar extinction coefficient of NADH at 340 nm; Sample volume pathlength (cm) for 96 well plate and 200 µl sample = 0.56). Lineweaver-Burk plot was used to determine Km and Vmax.

### DNA Cleavage Assays

Cleavage assays were performed as described [43]. Reaction was performed in a 25 µl mixture containing 10 mM Tris-HCl pH 7.5, 100 mM KCl, 5 mM MgCl2, 30 µg/ml BSA, 300 ng pUC119 plasmid, and 2–40 ng purified Topo II. Some reactions contained 10 mM EDTA. Various topoisomerase inhibitors were also added to the reactions to test the effect on cleavage activity of topoisomerases. After incubation at 37°C for 30 min, reaction was stopped by addition of 0.5% SDS, 10 mM EDTA, and 2 mg proteinase K and incubation at 37°C for 30 min. The resulting DNA was separated by electrophoresis on 1% agarose gels plus 25 µg/ml ethidium bromide.

### DNA Decatenation Assays

Decatenation assays were performed as described (www.topogen.com). Reaction was performed in a 20 ul mixture containing 40 mM Tris-HCl pH 7.8, 100 mM KCl, 18 mM MgCl2, 0.5 mM DTT, 0.5 mM EDTA, 1 mM ATP, 30 ug/ml BSA, 100 ng kDNA, and 40 ng purified TopoII. Various topoisomerase inhibitors were also added to the reactions to test the effect on decatenation activity of topoisomerases. After incubation at 37oC for 30 min, reaction was stopped by addition of 0.5% SDS, 10 mM EDTA, and 2 mg proteinase K and incubation at 37oC for 30 min. The resulting DNA was separated by electrophoresis on 1% agarose gels with ethidium bromide.

### ChIP Assays

The WB clone C6 cells were inoculated into encystation medium (5×10^7^ cells in 45 ml medium) and harvested after 24 h in encystation medium and washed in phosphate-buffered saline. ChIP was performed as described previously [24] with some modifications. Formaldehyde was then added to the cells in phosphate-buffered saline at a final concentration of 1%. Cells were incubated at room temperature for 15 min and reactions were stopped by incubation in 125 mM glycine for 5 min. After phosphate-buffered saline washes, cells were lysed in luciferase lysis buffer (Promega) and protease inhibitor (Sigma) and then vortexed with glass beads. The cell lysate was sonicated on ice and then centrifuged. Chromatin extract was incubated with protein G plus/protein A-agarose (Merck) for 1 h. After removal of protein G plus/protein A-agarose, the precleared lysates were incubated with 2 µg of anti-Myb2 antibody or preimmune serum for 2 h and then incubated with protein G plus/protein A-agarose (Merck) for 1 h. The beads were washed with low salt buffer (0.1% SDS, 1% Triton X-100, 2 mM EDTA, 20 mM Tris-HCl, pH 8.0, 150 mM NaCl) twice, high salt buffer (0.1% SDS, 1% Triton X-100, 2 mM EDTA, 20 mM Tris-HCl, pH 8.0, 500 mM NaCl) once, LiCl buffer (0.25 M LiCl, 1% Nonidet P-40, 1% sodium deoxycholate, 1 mM EDTA, 10 mM Tris-HCl, pH 8.0) once, and TE buffer (20 mM Tris-HCl, 1 mM EDTA, pH 8.0) twice. The beads were resuspended in elution buffer containing 50 mM Tris-HCl, pH 8.0, 1% SDS and 10 mM EDTA at 65°C for 4 h. To prepare DNA representing input DNA, 2.5% of precleared chromatin extract without incubation with anti-Topo II was combined with elution buffer. Eluted DNA was purified by the QIAquick PCR purification kit (Qiagen). Purified DNA was subjected to PCR reaction followed by agarose gel electrophoresis. Primers 18S5F and 18S5R were used to amplify the 18 S ribosomal RNA gene promoter as a control for our ChIP analysis. Primers topo II5F and topo II5R, cwp15F and cwp15R, cwp25F and cwp25R, cwp35F and cwp35R, myb25F and myb25R, and ran5F and ran5R were used to amplify *topo II*, *cwp1*, *cwp2*, *cwp3*, *myb2*, and *ran* gene promoters within the −200 to −1 region.

### Etoposide-Mediated Topoisomerase Immunoprecipitation Assays

The WB clone C6 cells were inoculated into encystation medium containing 400 µM etoposide (5×10^7^ cells in 45 ml medium) and harvested after 24 h and washed in phosphate-buffered saline. The assay was performed as described previously [38] with some modifications. Cells were lysed in lysis buffer (1% sarkosyl, 50 mM Tris-HC, pH 8.0, 5 mM EDTA, 1% Triton X-100, 120 mM NaCl) and protease inhibitor and then vortexed with glass beads. The cell lysate was treated with 5 M CsCl and sonicated on ice and then centrifuged. Chromatin extract in supernatant was incubated with protein G plus/protein A-agarose (Merck) for 1 h. After removal of protein G plus/protein A-agarose, the precleared lysates were incubated with 2 µg of anti-Topo II antibody or preimmune serum for 2 h and then incubated with protein G plus/protein A-agarose (Merck) for 1 h. The beads were washed with low salt buffer (50 mM Tris-HCl, pH 8.0, 5 mM EDTA, 1% Triton X-100, 150 mM NaCl) three times. The beads were resuspended in high salt elution buffer containing 50 mM Tris-HCl, pH 8.0, 5 mM EDTA, 1% Triton X-100, 500 mM NaCl. To prepare DNA representing input DNA, 2.5% of precleared chromatin extract without incubation with anti-Topo II was combined with high salt elution buffer. Eluted DNA was treated with 50 µg/ml RNase A and 200 µg/ml proteinase K and purified by the QIAquick PCR purification kit (Qiagen). Purified DNA was subjected to PCR reaction followed by agarose gel electrophoresis. Primers 18S5F and 18S5R were used to amplify the 18 S ribosomal RNA gene promoter as a control for our ChIP analysis. Primers topo II5F and topo II5R, cwp15F and cwp15R, cwp25F and cwp25R, cwp35F and cwp35R, myb25F and myb25R, and ran5F and ran5R were used to amplify *topo II*, *cwp1*, *cwp2*, *cwp3*, *myb2*, and *ran* gene promoters within the −200 to −1 region.

### Microarray Analysis

RNA was quantified by A260 nm by an ND-1000 spectrophotometer (Nanodrop Technology, USA) and qualitated by a Bioanalyzer 2100 (Agilent Technology) with an RNA 6000 Nano LabChip kit. RNA from the pPTopo II cell line was labeled by Cy5 and RNA from the 5′▵5N-Pac cell line was labeled by Cy3. In another experiment, RNA from the etoposide treated cells was labeled by Cy5 and RNA from non treated cells was labeled by Cy3. 0.5 µg of total RNA was amplified by a Low RNA Input Quick-Amp labeling kit (Agilent Technologies) and labeled with Cy3 or Cy5 (CyDye, Agilent Technologies) during the *in vitro* transcription process. 0.825 µg of Cy-labeled cRNA was fragmented to an average size of about 50–100 nucleotides by incubation with fragmentation buffer at 60°C for 30 minutes. Correspondingly fragmented labeled cRNA was then pooled and hybridized to a *G. lamblia* oligonucleotide microarray (Agilent Technologies, USA) at 65°C for 17 h. After washing and drying by nitrogen gun blowing, microarrays were scanned with an Agilent microarray scanner (Agilent Technologies) at 535 nm for Cy3 and 625 nm for Cy5. Scanned images were analyzed by Feature Extraction version 10.5.1.1 software (Agilent Technologies), and image analysis and normalization software was used to quantify signal and background intensity for each feature; data were substantially normalized by the rank consistency filtering LOWESS method. All data is MIAME compliant and that the raw data has been deposited in a MIAME (http://www.mged.org/Workgroups/MIAME/miame.html) compliant database (GEO) with accession number GSE39665.

## Results

### Identification and Characterization of *Topo II* Gene

**Figure 1:**
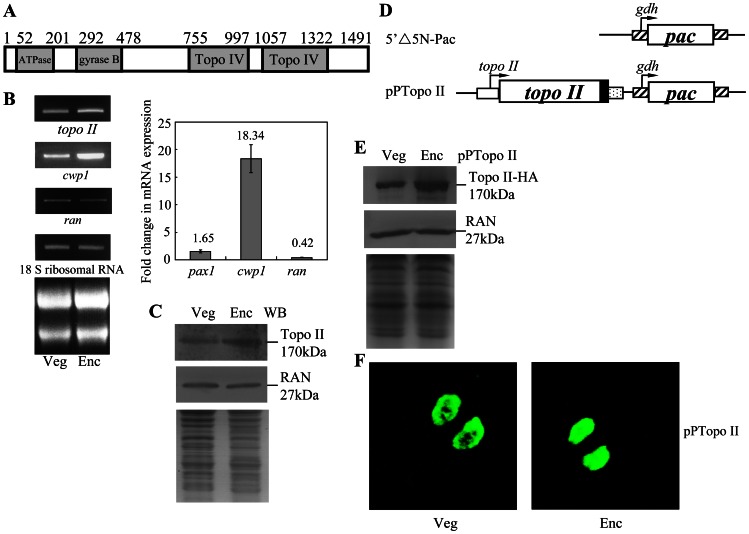
Analysis of *topo II* gene expression. (A) Schematic representation of the *Giardia* Topo II protein. The gray boxes indicate the ATPase, gyrase B, and Topo IV domains, as predicted by pfam (http://pfam.sanger.ac.uk/) [65]. (B) RT-PCR and quantitative real-time PCR analysis of *topo II* gene expression. RNA samples were prepared from *G. lamblia* wild type nontransfected WB cells cultured in growth (Veg, vegetative growth) or encystation medium and harvested at 24 h (Enc, encystation). RT-PCR was performed using primers specific for *topo II*, *cwp1*, *ran*, and 18 S ribosomal RNA genes. Ribosomal RNA quality and loading controls are shown in the bottom panel. Representative results are shown on the left. Real-time PCR was preformed using primers specific for *topo II*, *cwp1*, *ran*, and 18 S ribosomal RNA genes. Transcript levels were normalized to 18 S ribosomal RNA levels. -Fold changes in mRNAexpression are shown as the ratio of transcript levels in encysting cells relative to vegetative cells. Results are expressed as the means ± S.E. (error bars) of at least three separate experiments (right). (C) Topo II protein levels in different stages. The wild type nontransfected WB cells were cultured in growth (Veg, vegetative growth) or encystation medium for 24 h (Enc, encystation) and then subjected to SDS-PAGE and Western blot. The blot was probed by anti-Topo II and anti-RAN antibody. Representative results are shown. Equal amounts of protein loading were confirmed by SDS-PAGE and Coomassie Blue staining. (D) Diagrams of the 5′Δ5N-Pac and pPTopo II plasmid. The *pac* gene (open box) is under the control of the 5′- and 3′-flanking regions of the *gdh* gene (striated box). In construct pPTopo II, the *topo II* gene is under the control of its own 5′-flanking region (open box) and the 3′-flanking region of the *ran* gene (dotted box). The filled black box indicates the coding sequence of the HA epitope tag. (E) Topo II protein levels increased during encystation. The pPTopo II stable transfectants were cultured in growth (Veg, vegetative growth) or encystation medium for 24 h (Enc, encystation) and then subjected to SDS-PAGE and Western blot. HA-tagged Topo II protein was detected in the pPTopo II stable transfectants using an anti-HA antibody by Western blot analysis. Equal amounts of protein loading were confirmed by anti-Ran Western blot and SDS-PAGE with Coomassie Blue staining. (F) Nuclear localization of Topo II. The pPTopo II stable transfectants were cultured in growth (Veg, vegetative growth, left panel) or encystation medium for 24 h (Enc, encystation, right panel) and then subjected to immunofluorescence analysis using anti-HA antibody for detection. The product of pPTopo II localizes to the nuclei in both vegetative and encysting trophozoites.

To identify genes encoding novel topoisomerase proteins from *G. lamblia*, we searched the *G. lamblia* genome database (http://giardiadb.org/giardiadb/) [9], [60] with the keyword “topoisomerase” for annotated genes. This search detected four putative homologues for topoisomerases (data not shown). Only one of them is similar to type II topoisomerases, which was annotated as topoisomerase II (GenBank accession number **XP_001708897.1**, open reading frame 16975 in the *G. lamblia* genome database). Two of them are similar to type IA topoisomerases and one is similar to spo11 Type II DNA topoisomerases. We first focused on understanding the role of topoisomerase II (Topo II) in *Giardia*. This Topo II has been reported previously [56]. Comparison of genomic and cDNA sequences showed that the *topo II* gene contained no introns. The deduced *Giardia* Topo II protein contains 1491 amino acids with a predicted molecular mass of ∼164.01 kDa and a pI of 8.47. It has one putative DNA gyrase B domain (residues 337 to 436) and two domains of DNA gyrase/topoisomerase IV, A subunit (Topo IV domains) (residues 755 to 997 and 1057 to 1322) as predicted by Pfam (Fig. 1A and S1)(http://pfam.sanger.ac.uk/) [44], [65], [66]. An ATPase domain is present in *Giardia* Topo II (residues 52 to 201) and a conserved G-loop motif (GXXGXGXX) for ATP binding is also present in *Giardia* Topo II (residues 140 to 147, GRNGYGAK) (Fig. 1A and S1) [56]. Sequence alignment shows that *Giardia* Topo II is moderately similar to the human topoisomerase IIα/β and the size of *Giardia* Topo II (1491 amino acids) is smaller than that of human topoisomerase IIα/β (1531/1621 amino acids) (Fig. S1). *Giardia* Topo II also has a conserved Tyr (residue 847), corresponding to the catalytic important Tyr of human topoisomerase IIα and IIβ (residues 805 and 821, respectively) (Fig. S1) [67].

The sequence of the ATPase, DNA gyrase B, and Topo IV domains has moderate similarity to those of the human topoisomerase IIα/β (Fig. S1). Two insertions are present in the DNA gyrase B domain of *Giardia* Topo II (Fig. S1). Giardial genes frequently have unique amino acid inserts [4]. The full-length of *Giardia* Topo II has 28% (27%) identity and 41% (39%) similarity to that of human topoisomerase IIα/β (calculated from Fig. S1). The *Giardia* Topo II has two Topo IV domains because the residues 998 to 1056 were not predicted as Topo IV domain by pfam analysis. The two Topo IV domains of *Giardia* Topo II (residues 755 to 1322) have 30% identity and 45% similarity to the Topo IV domain of the human topoisomerase IIα/IIβ (residues 713–1171 or 729–1184) (calculated from Fig. S2). Several insertions are present in the Topo IV domains of *Giardia* Topo II (Fig. S2). The C terminal region of *Giardia* Topo II has no apparent functional motif and has lower similarity to that of the human topoisomerase IIα/β (Fig. S1). The sequence variation may help design therapeutic drugs for giardiasis. A neighbor-joining phylogenetic tree obtained from the alignment of the Topo II proteins from various organisms revealed similarity between *Giardia* Topo II, *Trichomonas* Topo II and *Entamoeba* Topo II (Fig. S3).

### Encystation-Induced Expression of the *Topo II* Gene

RT-PCR and quantitative real-time PCR analysis of total RNA showed that the *topo II* transcript was present in vegetative cells and increased by ∼1.65-fold in 24 h encysting cells (Fig. 1B). As controls, we found that the mRNA levels of the *cwp1* and *ran* genes increased and decreased significantly during encystation, respectively (Fig. 1B). The products of the *cwp1* and *ran* genes are the component of the cyst wall and the *ras*-related nuclear protein [11], [17]. To determine the expression of the Topo II protein, we generated an antibody specific to the full-length Topo II. Western blot analysis confirmed that this antibody recognized Topo II at a size of ∼170 kDa (Fig. 1C), which was almost matched to the predicted molecular mass of Topo II (∼164.01 kDa). Topo II was expressed in vegetative cells and its levels increased significantly during encystation (Fig. 1C). As a control, the levels of the giardial RAN protein (∼27 kDa) decreased slightly during encystation (Fig. 1C). The preimmune serum did not detect any bands at a size of ∼170 kDa (data not shown).

### Nuclear Localization of the Topo II Protein

To determine the role of Topo II protein, we prepared construct pPTopo II, in which the *topo II* gene is controlled by its own promoter and contains an HA epitope tag at its C terminus (Fig. 1D) and stably transfected it into *Giardia*. Similar to the expression pattern of the endogenous Topo II protein, the levels of the Topo II-HA protein increased significantly during encystation (Fig. 1E). The HA-tagged Topo II was detected exclusively in the nuclei during vegetative growth and encystation (Fig. 1F), indicating that Topo II is a nuclear protein in *Giardia*. As a negative control, there was no staining for anti-HA antibody detection in the 5′Δ5N-Pac cell line, which expressed only the puromycin selection marker (Fig. 1D and data not shown).

### Change of Localization of the Topo II Mutants

**Figure 2:**
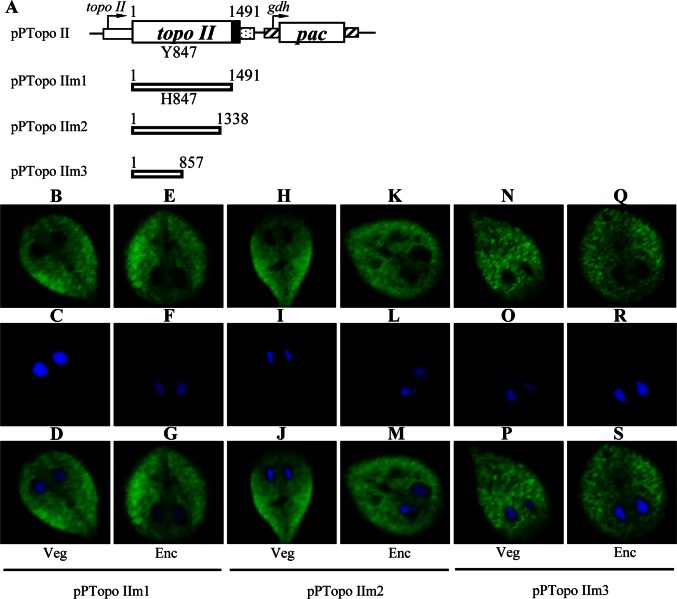
Localization of Topo II mutants. (A) Diagrams of the pPTopo II and pPTopo IIm1-3 plasmids. The expression cassettes of the *pac* gene and *topo II* gene are the same as in Fig. 1D. The residue Tyr 847 (Y847), which is important for Topo II activity, is mutated to His (H847) in Topo IIm1. Topo IIm2 does not contain the C terminal 153 amino acids (deletion of residues 1339–1491). Topo IIm3 does not contain most of the Topo IV domain (deletion of residues 858–1491). The *topo II* gene was mutated and subcloned to replace the wild-type *topo II* gene in the backbone of pPTopo II (Fig. 1D), and the resulting plasmids pPTopo IIm1-3 were transfected into *Giardia*. (B) Immunofluorescence analysis of Topo IIm1-3 distribution. The pPTopo IIm1-3 stable transfectants were cultured in growth (Veg, vegetative growth) or encystation medium for 24 h (Enc, encystation) and then subjected to immunofluorescence analysis using anti-HA antibody for detection. The products of pPTopo IIm1-3 localized to the cytoplasm in both vegetative and encysting trophozoites (panels B-S). Panels C, F, I, L, O, and R show the DAPI staining of cell nuclei. Panels D, G, J, M, P, and S are the merged images of B and C, E and F, H and I, K and L, N and O, Q and R, respectively.

We also performed mutation analysis to understand the role of Topo II. It is known that human topoisomerases II can create a transient double stranded DNA break by transesterification of an important Tyr of the cleavage domain and a DNA phosphodiester bond [33], [34]. We tried to understand whether Tyr 847 of Topo II, which corresponds to Tyr 805 of the human topoisomerase IIα, is also important for its activity (Fig. S1). We found that mutation of the Topo II Tyr 847 to His resulted in loss of nuclear localization in both vegetative and encysting cells (Topo IIm1, Fig. 2A–G), suggesting that the Tyr 847 residue may play an important role in the exclusive nuclear localization. We also found that deletion of the C-terminal 153 amino acids (residues 1339–1491, pPTopo IIm2, Fig. 2A and H–M) resulted in a significant decrease of nuclear localization. Deletion of the C-terminal region containing the Topo IV domain (residues 858–1491, pPTopo IIm3, Fig. 2A and N-S) resulted in a significant decrease of nuclear localization. Topo IIm1, Topo IIm2, and Topo IIm3 localized in the cytoplasm of both vegetative and encysting cells (Fig. 2B-S).

### Overexpression of Topo II Induced the Expression of *Cwp1-3* and *Myb2* Genes

**Figure 3:**
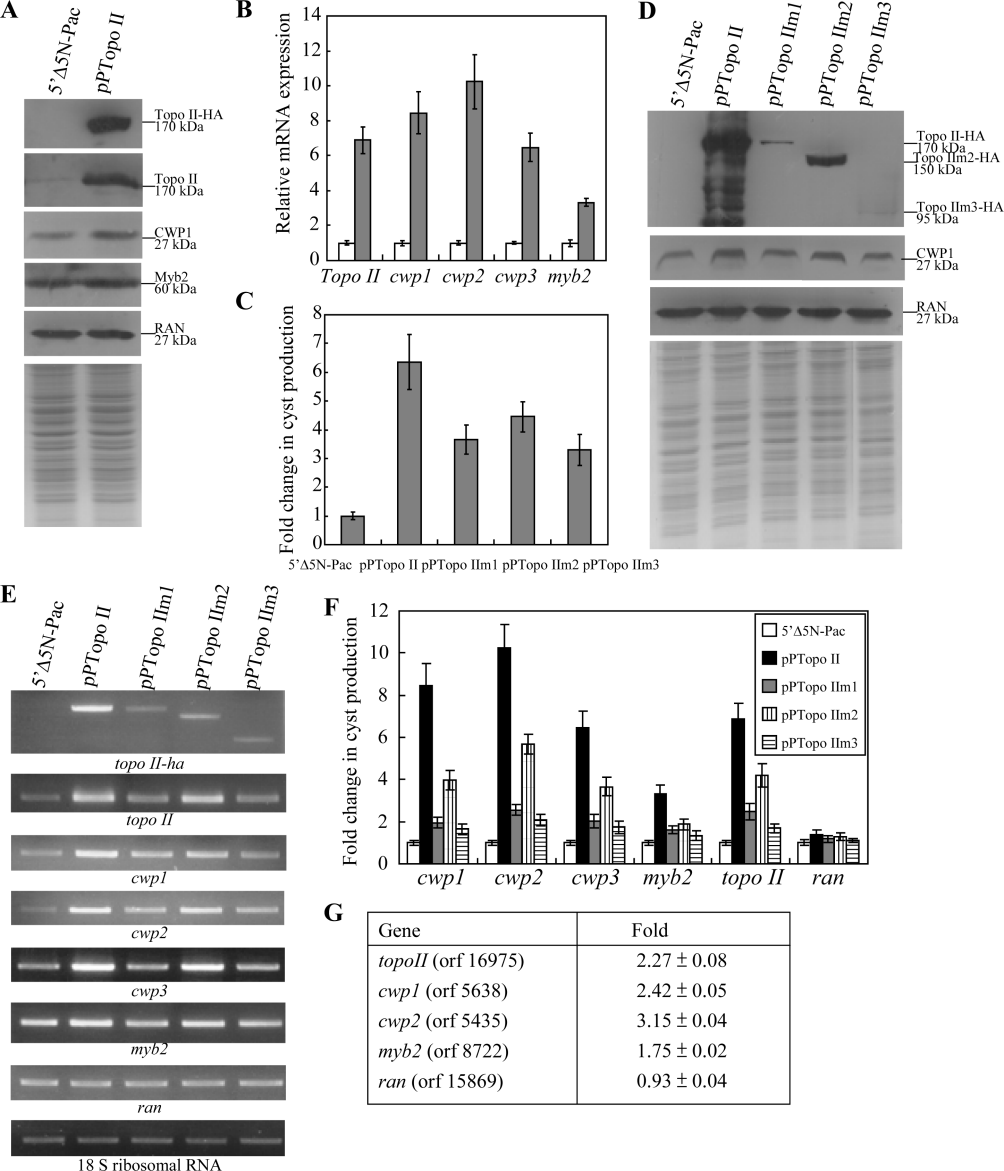
Induction of *cwp1-3* and *myb2* gene expression in the Topo II overexpressing cell line. (A) Overexpression of Topo II increased the levels of CWP1 protein. The 5′Δ5N-Pac and pPTopo II stable transfectants were cultured in growth medium and then subjected to SDS-PAGE and Western blot. The blot was probed by anti-HA, anti-Topo II, anti-CWP1, anti-Myb2, and anti-Ran antibodies. Equal amounts of protein loading were confirmed by SDS-PAGE and Coomassie Blue staining. Representative results are shown. (B) Quantitative real-time PCR analysis of gene expression in the Topo II -overexpressing cell line. The 5′▵5N-Pac and pPTopo II stable transfectants were cultured in growth medium and then subjected to quantitative real-time PCR analysis. Real-time PCR was preformed using primers specific for *topo II*, *cwp1*, *cwp2*, *cwp3*, *myb2*, *ran*, and 18 S ribosomal RNA genes. Similar mRNA levels of the *ran* and 18 S ribosomal RNA genes for these samples were detected (data not shown). Transcript levels were normalized to 18 S ribosomal RNA levels. Fold changes in mRNA expression are shown as the ratio of transcript levels in the pPTopo II cell line relative to the 5′▵5N-Pac cell line. Results are expressed as the means ± S. E. of at least three separate experiments. (C) Cyst count. The 5′Δ5N-Pac, pPTopo II, pPTopo IIm1, pPTopo IIm2, and pPTopo IIm3 stable transfectants were cultured in growth medium and then subjected to cyst count as described under “Experimental Procedures”. The sum of total cysts is expressed as relative expression level over control. Values are shown as means ± S. E. (D) Analysis of Topo II mutants. The 5′Δ5N-Pac, pPTopo II, pPTopo IIm1, pPTopo IIm2, and pPTopo IIm3 stable transfectants were cultured in growth medium and then subjected to SDS-PAGE and Western blot. The blot was probed by anti-HA, anti-CWP1, and anti Ran antibodies. Equal amounts of protein loading were confirmed by SDS-PAGE and Coomassie Blue staining. Representative results are shown. (E) RT-PCR analysis of gene expression in the Topo II- and Topo II mutants- overexpressing cell lines. The 5′Δ5N-Pac, pPTopo II, pPTopo IIm1, pPTopo IIm2, and pPTopo IIm3 stable transfectants were cultured in growth medium and then subjected to RT-PCR analysis. PCR was performed using primers specific for *topo II-ha*, *topo II*, *cwp1*, *cwp2*, *cwp3*, *myb2*, *ran*, and 18 S ribosomal RNA genes. (F) Quantitative real-time PCR analysis of gene expression in the Topo II and Topo IIm1-3 overexpressing cell lines. Real-time PCR was performed using primers specific for *topo II*, *cwp1*, *cwp2*, *cwp3*, *myb2*, *ran*, and 18 S ribosomal RNA genes. Similar mRNA levels of the 18 S ribosomal RNA genes for these samples were detected. Transcript levels were normalized to 18 S ribosomal RNA levels. Fold changes in mRNA expression are shown as the ratio of transcript levels in the pPTopo II or pPTopo IIm1-3 cell line relative to the 5′Δ5N-Pac cell line. Results are expressed as the means ± standard error of at least three separate experiments. (G) Microarray analysis. Microarray data were obtained from the 5′Δ5N-Pac and pPTopo II cell lines during vegetative growth. Fold-changes are shown as the ratio of transcript levels in the pPTopo II cell line relative to the 5′Δ5N-Pac cell line. Results are expressed as the mean ± S. E. of at least three experiments.

To study the role of Topo II in *G. lamblia*, we expressed *topo II* by its own promoter (pPTopo II; Fig. 1C) and observed its gene expression. The Topo II-HA protein (∼170 kDa) was expressed in the pPTopo II stable cell line but not in the control cell line (5′Δ5N-Pac) (Fig. 1D) as detected by anti-HA antibody in Western blots (Fig. 3A). Overexpression of Topo II in the pPTopo II cell line also can be confirmed by the anti-Topo II antibody (Fig. 3A). We found that Topo II overexpression resulted in a significant increase of the CWP1 (2.1-fold) and Myb2 (∼1.8-fold) protein levels during vegetative growth (Fig. 3A). As a control, similar levels of intensity of the giardial RAN protein (∼27 kDa) were detected by anti-RAN antibody (Fig. 3A). Quantitative real time PCR analysis showed that the mRNA levels of the endogenous *topo II* plus vector expressed *topo II* in the Topo II overexpressing cell line increased by ∼6.88-fold (*p*<0.05) relative to the 5′Δ5N-Pac control cell line (Fig. 3B). The mRNA levels of the endogenous *cwp1-3* and *myb2* genes in the Topo II overexpressing cell line increased by ∼3.32 to ∼10.24-fold (*p*<0.05) relative to the 5′Δ5N-Pac control cell line (Fig. 3B). Similar mRNA levels of the *ran* and 18 S ribosomal RNA genes were detected (data not shown). Similar results were obtained during encystation (Fig. S4). Our results suggest that overexpression of Topo II can induce expression of the *cwp1-3* and *myb2* genes.

We further investigated the effect of giardial Topo II on cyst formation. In previous studies, we have found that some *G. lamblia* trophozoites may undergo spontaneous differentiation [59]. We obtained consistent cyst count data for vegetative *G. lamblia* cultures during growth to stationary phase (∼4800 cysts/ml for 5′Δ5N-Pac cell line) [59]. In this study, we found that the cyst number in the Topo II overexpressing cell line increased by ∼6.35-fold (*p*<0.05) relative to the control cell line, indicating that the overexpressed Topo II can increase cyst formation (Fig. 3C). Similar results were obtained during encystation (Fig. S4). The results suggest that overexpression of Topo II can increase cyst formation.

To further understand the function of giardial Topo II, we analyzed the effect of mutation of Topo II. Topo IIm1, Topo IIm2, and Topo IIm3 have a cytoplasmic localization and can not enter nuclei (Fig. 2). We found that the levels of Topo IIm1, Topo IIm2, and Topo IIm3 decreased significantly compared with that of wild type Topo II during vegetative growth in anti-HA Western blots (m1, m2, m3 to ∼14%, ∼51, or ∼08% of the wild type value, respectively) (Fig. 3D). We also found that levels of the CWP1 protein decreased significantly in the Topo IIm1, Topo IIm2, and Topo IIm3 overexpressing cell lines relative the wild type Topo II overexpressing cell line (m1, m2, m3 to ∼52%, ∼78%, or ∼55% of the wild type value, respectively)(Fig. 3D). Topo IIm1, Topo IIm2, and Topo IIm3 did not localize to nuclei (Fig. 2), suggesting of their potential functional loss. As a control, similar levels of intensity of the giardial RNA protein (∼27 kDa) were detected by anti-RAN antibody (Fig. 3D). We further analyzed whether the transcript levels of the Topo IIm1, Topo IIm2, and Topo IIm3 were changed. As shown by RT-PCR and quantitative real-time PCR analysis, the levels of HA-tagged *Topo IIm1*, *Topo IIm2*, and *Topo IIm3* mRNA decreased significantly compared with that of wild type HA-tagged *Topo II* during vegetative growth (m1, m2, m3 to ∼36%, ∼61%, or ∼24% of the wild type value, respectively) (Fig. 3E). We also found that the levels of *cwp1-3* and *myb2* mRNA decreased significantly in the Topo IIm1, Topo IIm2, and Topo IIm3 overexpressing cell lines relative to the wild type Topo II overexpressing cell line (to between ∼19% and 56% of the wild type value)(Fig. 3E). Similar mRNA levels of the *ran* and 18 S ribosomal RNA genes were detected (Fig. 3E). We also tried to detect sense transcript and found similar results (Fig. S5A). The levels of cyst formation decreased significantly in the Topo IIm1, Topo IIm2, and Topo IIm3 overexpressing cell lines relative to the wild type Topo II overexpressing cell line (to between ∼51% and 70% of the wild type value)(Fig. 3C). Similar results were obtained during encystation (Fig. S4). The results suggest a decrease of encystation-induced activity of Topo IIm1, Topo IIm2, and Topo IIm3.

Oligonucleotide microarray assays confirmed up-regulation of *cwp1*, *cwp2*, and *myb2* expression in the Topo II overexpressing cell line ∼1.75 to ∼3.15-fold of the levels in the control cell line (Fig. 3G). Expression levels of the *cwp3* gene in the Topo II overexpressing cell line only increased insignificantly (∼1.18-fold; data not shown). Similar mRNA levels of the *ran* gene were detected (Fig. 3G). Oligonucleotide microarray assays identified up-regulation of several *vsp* genes in the Topo II overexpressing cell line (Table S2). We found that 95 and 20 genes were significantly up-regulated (>2-fold) and down-regulated (<1/2)(*p*<0.05) in the Topo II overexpressing cell line relative to the vector control, respectively (Table S2). Expression levels of the *topo II* gene in the Topo II overexpressing cell line increased by ∼2.27-fold (*p*<0.05)(Fig. 3G and Table S2).

### Topo II Has DNA Cleavage Activity

**Figure 4:**
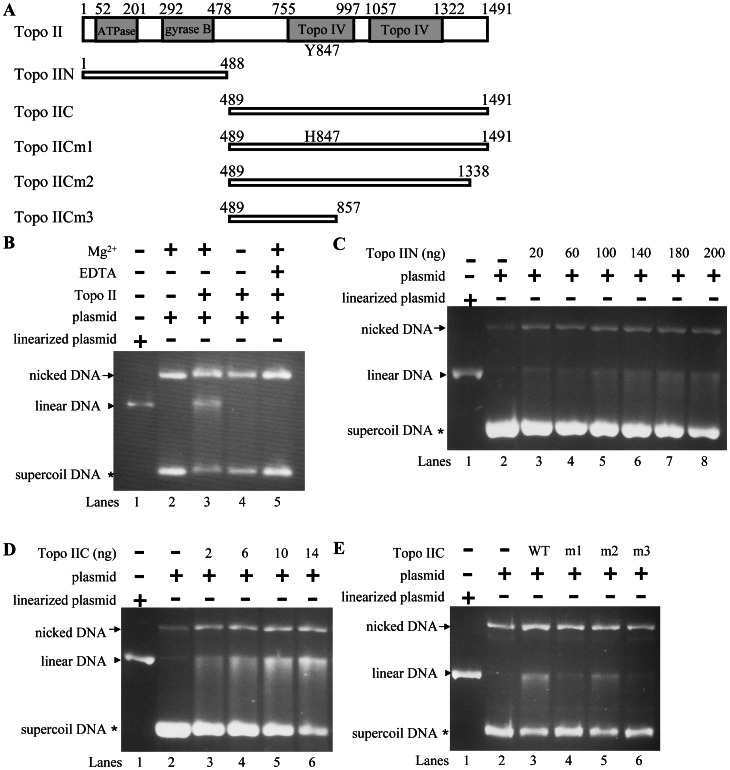
Cleavage activity of Topo II. (A) Schematic representation of the *Giardia* Topo II protein. The gray boxes indicate the ATPase, gyrase B, and Topo IV domains, as predicted by pfam (http://pfam.sanger.ac.uk/) [65]. (B) DNA cleavage activity of Topo II. DNA cleavage assays were performed with purified recombinant Topo II and pUC119 plasmid (3.1 kb). Components in the reaction are indicated above the lanes. Typically, 2 ng Topo II was mixed with 300 ng plasmid DNA. Some reaction mixtures contain 5 mM magnesium II ion or 10 mM EDTA, as indicated. Linearized plasmid is included as a size marker. (C) DNA cleavage activity of Topo IIN mutant. DNA cleavage assays were performed with pUC119 plasmid and purified recombinant Topo IIN in a buffer containing 5 mM magnesium II ion. Components in the reaction are indicated above the lanes. Topo IIN as indicated levels was mixed with 300 ng plasmid DNA. Linearized plasmid is included as a size marker. (D) DNA cleavage activity of Topo IIC mutant. DNA cleavage assays were performed with pUC119 plasmid and purified recombinant Topo IIC in a buffer containing 5 mM magnesium II ion. Components in the reaction are indicated above the lanes. Topo IIC as indicated levels was mixed with 300 ng plasmid DNA. Linearized plasmid is included as a size marker. (E) DNA cleavage activity of Topo IICm1-3 mutants. DNA cleavage assays are performed with pUC119 plasmid and purified recombinant Topo IIC and Topo IICm1-3 in a buffer containing 5 mM magnesium II ion. Components in the reaction are indicated above the lanes. Typically, 10 ng Topo IIC or Topo IICm1-3 was mixed with 300 ng plasmid DNA. Linearized plasmid is included as a size marker.

Type II topoisomerases have ability to cleave double stranded DNA [33], [34]. To test DNA cleavage activity of Topo II, we expressed Topo II in *E. coli* and purified it to >95% homogeneity, as assessed in a silver-stained gel (Figs. 4A and Fig. S5B). We performed DNA cleavage assays with purified recombinant Topo II and pUC119 plasmid. As shown in Fig. 4B, Topo II has a significant DNA cleavage activity. This activity is dependent on the presence of magnesium II ion (Fig. 4B, lane 3). Addition of EDTA disrupted the cleavage activity of Topo II (Fig. 4B, lane 5). The results indicate that Topo II may function as a type II topoisomerase in *Giardia* and magnesium II ion is required for full activity of Topo II. In normal condition of the cleavage assays, proteinase K was included to stop the reaction for removing Topo II of the cleavage complex (Fig. 4B). When proteinase K was not included in the stop reaction of the cleavage assays, the Topo II-DNA cleavage complex can not be resolved in the gel (Fig. S5C, lane 3), suggesting the presence of the Topo II-DNA cleavage complex.

To understand which regions are important for cleavage activity, specific Topo II mutants were expressed in *E. coli*, purified, and tested for their DNA binding activity (Fig. 4A). We found that deletion of the C terminal region of Topo II resulted in a loss of cleavage activity (Topo IIN) (Fig. 4C, lanes 3–8). The cleavage activity of the Topo IIN mutant was very low even with addition of 200 ng of Topo IIN in the reaction (Fig. 4C, lane 8). Deletion of the N terminal region of Topo II (Topo IIC) did not affect the cleavage activity and the cleavage activity was Topo IIC dose dependent (Fig. 4D, lanes 3–6). We further created three mutants that are based on the Topo IIC backbone and contain a mutation of the catalytic important Tyr 847 (Topo IICm1), a deletion of C terminal 153 amino acids (residues 1339-1491, Topo IICm2), or a deletion of the C-terminal region containing the Topo IV domain (residues 858–1491, Topo IICm3) (Fig. 4A). We found a significant loss of cleavage activity of Topo IICm1 and Topo IICm3 and no significant change of cleavage activity of Topo IICm2 (Fig. 4E, lanes 3–6). Similar levels of wild type Topo II and Topo II mutants were added to the reaction mixtures (data not shown).

### Topo II Has ATPase Activity

**Figure 5:**
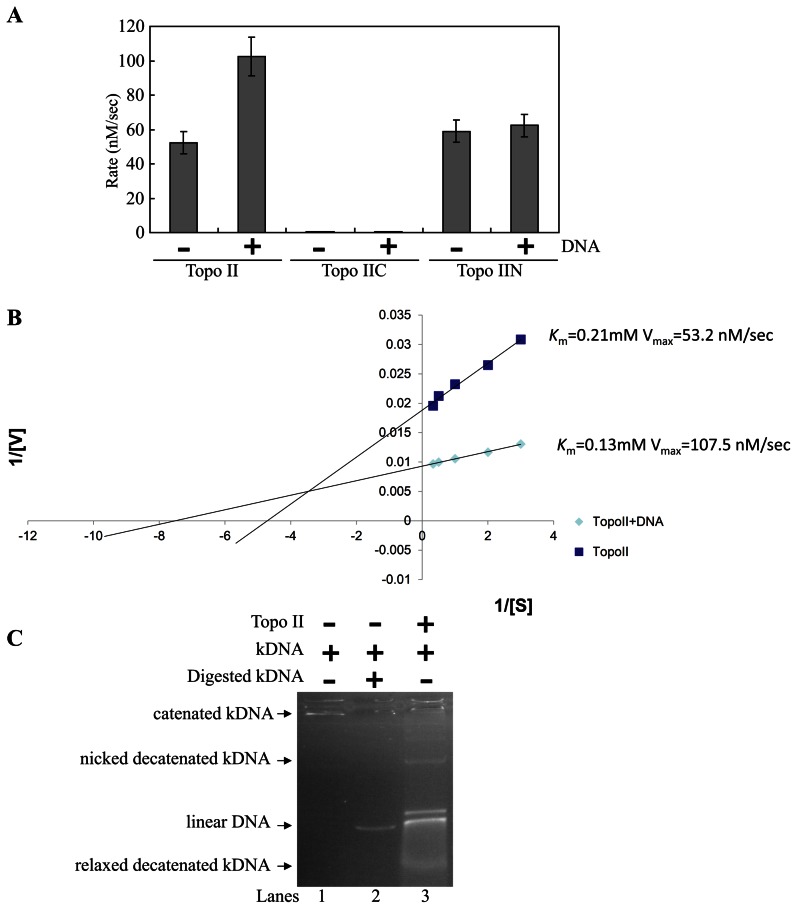
Analysis of the ATPase activity of Topo II. (A) ATPase activity of the Topo II, Topo IIC, and Topo IIN proteins. ATPase assays were performed with 8 ng purified recombinant Topo II, 80 ng Topo IIC, or 80 ng Topo IIN as described in Fig. 4A at 3 mM ATP concentration in the absence or presence of 300 ng plasmid DNA (pGEM-Teasy vector, 3.6 kb). The data shown are means ± S.E. for three independent experiments. (B) ATPase activity of Topo II. ATPase assays were performed with 8 ng Topo II in the absence and in the presence of 300 ng plasmid DNA (pGEM-Teasy vector). Maximal reaction rate (Vmax) and Km were estimated from Lineweaver-Burk plots. (C) Decatenation activity of Topo II. Decatenation assays were performed with 100 ng kDNA and 40 ng purified TopoII. Linear kDNA was produced by incubating with an restriction enzyme (lane 2).

We also performed ATPase assays with purified recombinant Topo II. As shown in Fig. 5A, Topo II has a significant ATPase activity. Addition of plasmid DNA increased the ATPase activity of Topo II, suggesting that this activity is dependent on the presence of DNA (Fig. 5A). The results indicate that Topo II may function as a type II topoisomerase in *Giardia* and that DNA is required for full activity of Topo II.

To understand which regions are important for ATPase activity, specific Topo II mutants were expressed in *E. coli*, purified, and tested for their DNA binding activity (Fig. 4B). We found that deletion of the N terminal region of Topo II resulted in a complete loss of ATPase activity (Topo IIC) (Fig. 5A). Deletion of the C terminal region of Topo II did not affect the ATPase activity (Topo IIN) (Fig. 5A). However, the lack of C terminal region resulted in a loss of DNA dependent ATPase activity (Topo IIN+DNA)(Fig. 5A). We further created Lineweaver-Burk plot to determine Km and Vmax for Topo II ATPase activity. The Km value for Topo II is 0.21 mM (Fig. 5B). The Km value decreased to 0.13 mM with the addition of DNA, suggesting an increase of affinity for Topo II and substrate by DNA addition (Fig. 5B). The Vmax value for Topo II is 53.2 nM/sec (Fig. 5B). The Vmax value increased to 107.5 nM/sec with the addition of DNA (Fig. 5B). The results suggest that the ATPase activity of Topo II can be induced by the presence of DNA.

We also found that Topo II has decatenation activity that produced nicked decatenated kDNA and relaxed decatenated kDNA (Fig. 5C), suggesting that Topo II has type II topoisomerase activity.

### Topo II Has DNA Binding Activity

**Figure 6:**
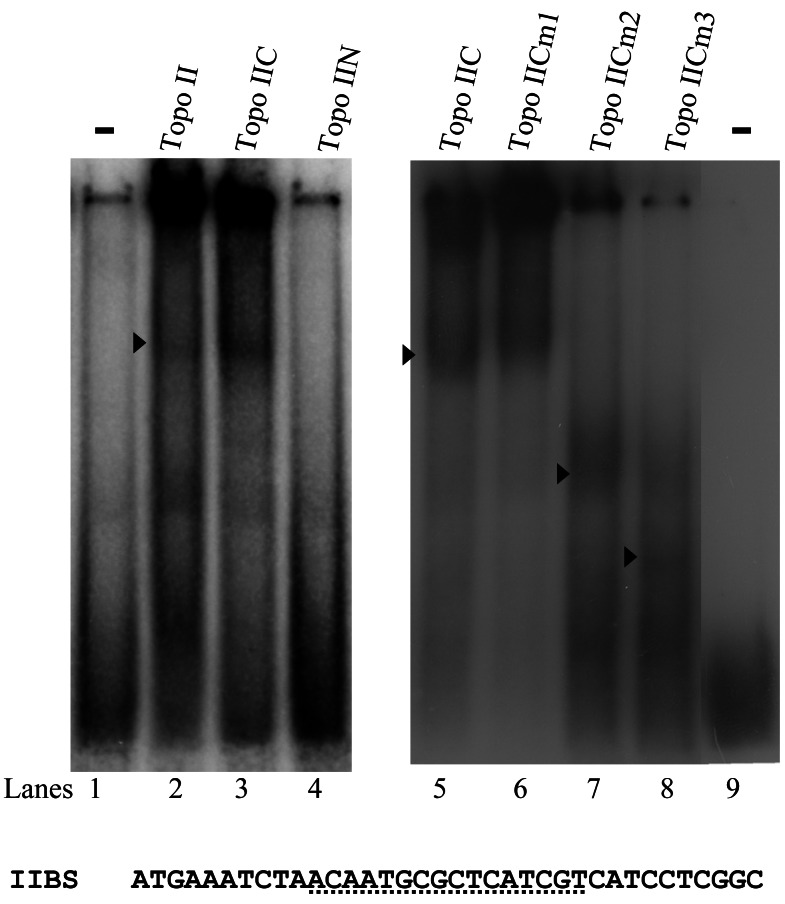
Decrease of DNA binding activity of Topo II mutants. Electrophoretic mobility shift assays were performed using purified Topo II, Topo IIC, Topo IIN, and Topo IICm1-3 as described in Fig. 4A and the ^32^P-end-labeled oligonucleotide probe IIBS. The arrowheads indicate the shifted complexes. The binding sequence of human topoisomerase II is indicated by a dotted line in the IIBS probe [68].

We further tested DNA-binding activity of Topo II. Electrophoretic mobility shift assays were performed with the purified Topo II protein and double-stranded DNA sequences from the 5′-flanking region of *cwp* genes and human topoisomerase II binding sequence (IIBS) [68]. Incubation of a labeled double-stranded DNA probe, IIBS, with Topo II resulted in the formation of retarded bands (Fig. 6, lane 2). To understand which regions are important for DNA binding, specific Topo II mutants were tested for their DNA binding activity. Similar levels of wild type Topo II and its mutants were added to the binding reaction mixtures (data not shown). We found that deletion of the C terminal region of Topo II resulted in a complete loss of DNA binding activity to the IIBS probe (Topo IIN) (Fig. 6, lane 4). Deletion of the N terminal region of Topo II did not change the DNA binding activity (Topo IIC) (Fig. 6, lane 3). Three mutants based on the Topo IIC were also tested for DNA binding activity. There was no significant change of the DNA binding activity of Topo IICm1 (Fig. 6, lane 6). We found a slight decrease of DNA binding activity of Topo IICm2 and a significant decrease of DNA binding activity of Topo IICm3 (Fig. 6, lanes 7 and 8). We also found a formation of the shifted bound form of Topo IICm2 and Topo IICm3, which is matched to their characteristics as they are deletion mutants (Fig. 6, lanes 7 and 8).

**Figure 7:**
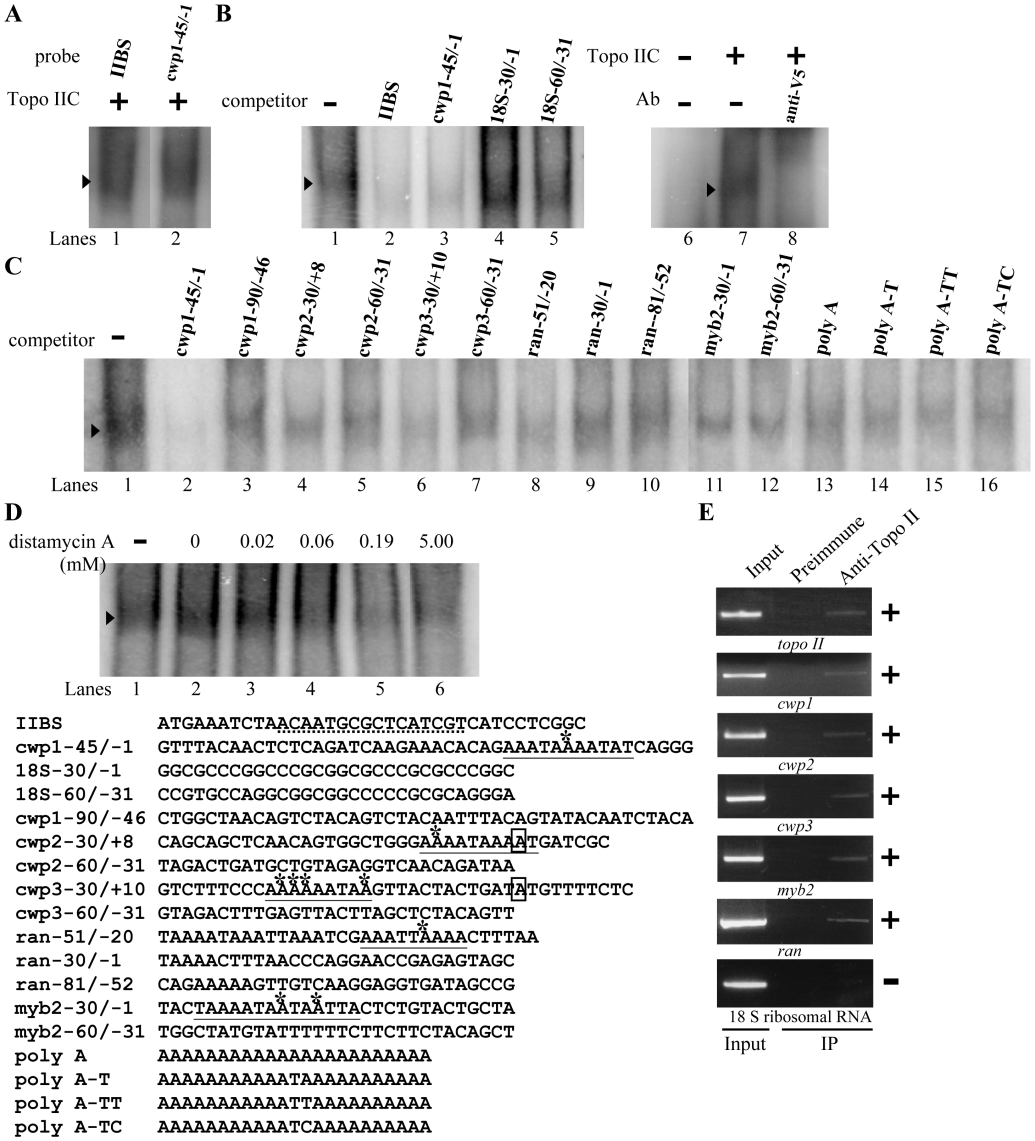
DNA binding ability of Topo IIC revealed by electrophoretic mobility shift assays. (A) Detection of Topo IIC binding sites. Electrophoretic mobility shift assays were performed using purified Topo IIC and the ^32^P-end-labeled oligonucleotide probe IIBS or cwp1-45/-1 (−45 to −1 relative to the translation start site of the *cwp1* gene). Components in the binding reaction mixtures are indicated above the lanes. The arrowhead indicates the shifted complex. (B) Binding specificity of the Topo IIC. The Topo IIC binding specificity was confirmed by competition and supershift assays. Some reaction mixtures contained 200-fold molar excess of cold oligonucleotides or 0.8 µg of anti-V5-horseradish peroxidase antibody as indicated above the lanes. (C) Detection of Topo IIC binding sites in multiple promoters. Purified Topo IIC and ^32^P labeled oligonucleotide probe IIBS was used in reaction mixtures. Reaction mixtures also contained 200-fold molar excess of cold oligonucleotides as indicated above the lanes. The transcription start sites of the *cwp1*, *cwp2*, *cwp3*, and *myb2* genes determined from 24-h encysting cells are indicated by asterisks [17], [18], [19], [22]. The AT-rich initiator elements spanning the transcription start sites are underlined. The translation start sites of the *cwp2* and *cwp3* genes are framed. “18 S” represents 18 S ribosomal RNA. (D) Effect of distamycin A on the binding of Topo IIC to DNA. ^32^P-end-labeled IIBS probe was incubated with Topo IIC in the absence (lane 1) or presence of distamycin A (lanes 3–6). Distamycin A was dissolved in Me2SO. Adding Me2SO to the reaction mix did not decrease the Topo IIC binding activity (lane 2). (E) Recruitment of Topo II to the *cwp* and *myb2* promoters. The nontransfected WB cells were cultured in encystation medium containing 400 µM etoposide for 24 h and then subjected to etoposide-mediated topoisomerase immunoprecipitation assays. Anti-Topo II was used to assess binding of Topo II to endogenous gene promoters. Preimmune serum was used as a negative control. Immunoprecipitated chromatin was analyzed by PCR using primers that amplify the 5′-flanking region of specific genes. At least three independent experiments were performed. Representative results are shown. Immunoprecipitated products of Topo II yield more PCR products of *topo II*, *cwp1*, *cwp2*, *cwp3*, *myb2*, and *ran* promoters, indicating that Topo II was bound to these promoters (+). The 18 S ribosomal RNA gene promoter was used as a negative control for our etoposide-mediated topoisomerase immunoprecipitation assays (−).

Topo IIC was also shown to bind to the *cwp1* promoter (cwp1-45/-1) (Fig. 7A, lane 2). cwp1-45/-1 is the region from −45 to −1 bp relative to the translation start site of the *cwp1* gene. Incubation of the GC rich probes 18S-30/-1 and 18S-60/-31 (Fig. 7) with Topo IIC did not form any retarded bands (data not shown), suggesting that Topo IIC did not bind to GC rich sequence. The binding specificity was confirmed by competition and supershift assays (Fig. 7B). The formation of the shifted IIBS bands was almost totally competed by a 200-fold molar excess of unlabeled IIBS and cwp1-45/-1, but not by the same excess of a nonspecific competitor, 18S-30/-1 and 18S-60/-31 (Fig. 7B, lanes 2–5). Topo IIC bound to IIBS could be supershifted by an anti-V5-horseradish peroxidase antibody that recognized the purified Topo IIC protein (Fig. 7B, lane 8). The results suggest that *Giardia* Topo II can bind the *cwp1* promoter (−45/−1 region).

Competition analysis was used to test the binding affinity of DNA fragments for Topo IIC. DNA fragments with the higher binding affinity for Topo IIC are more effective in the competition analysis. Topo IIC was also shown to bind weakly to the cwp1-90/-46 (Fig. 7C, lane 3). We also tested whether Topo IIC binds to the 5′-flanking region of other genes. We found that the cwp2-30/+8, cwp3-30/+10, and ran-51/-20 sequences were more effective in the competition analysis, suggesting that Topo IIC bound strongly to these sequences (Fig. 7C, lanes 4, 6, and 8). In addition, the cwp2-60/-31, cwp3-60/-31, ran-30/-1, ran-81/-52, myb2-60/-31, and myb2-30/-1 sequences had weak competition ability, suggesting that Topo IIC bound weakly to these sequences (Fig. 7C, lanes 6, 7, 9, 10, 11 and 12). The results suggest that these specific promoters may contain putative Topo IIC binding sequences, and competitive effectiveness correlated with the number of AT-rich sequences present in the fragments. We also tested whether Topo IIC binds to specific AT-rich sequences. Interestingly, Topo IIC also bound to a poly(A) sequence and poly(A) sequence with a T, TT, or TC insertion (Fig. 7C, lanes 13–16).

To investigate how Topo IIC binds DNA, we used distamycin A, which binds to the DNA minor groove, as a competitive inhibitor of Topo IIC binding [69]. As shown in Fig. 7D, the binding of Topo IIC to DNA decreased with increasing concentrations of distamycin A. However, the binding was not completely inhibited at concentrations ∼5 mM, suggesting that Topo IIC may bind to both major and minor grooves.

### Recruitment of Topo II to the *Topo II*, *Cwp1*-*3* and *Myb2* Promoters

We further used etoposide-mediated topoisomerase immunoprecipitation assay [38], a method similar to ChIP assays to study the association of Topo II with specific promoters. Addition of etoposide may increase the cleavage complex formation and thereby increase ChIP sensitivity [38]. We found that Topo II was associated with its own promoter and the *cwp1*, *cwp2*, *cwp3*, *myb2*, and *ran* promoters during encystation (Fig. 7E). However, Topo II was not associated with the 18 S ribosomal RNA gene promoter which has no Topo II binding site in the <200 bp 5′-flanking region (Fig. 7E)(data not shown).

### Regulation of *Topo II* Gene Expression by Myb2

**Figure 8:**
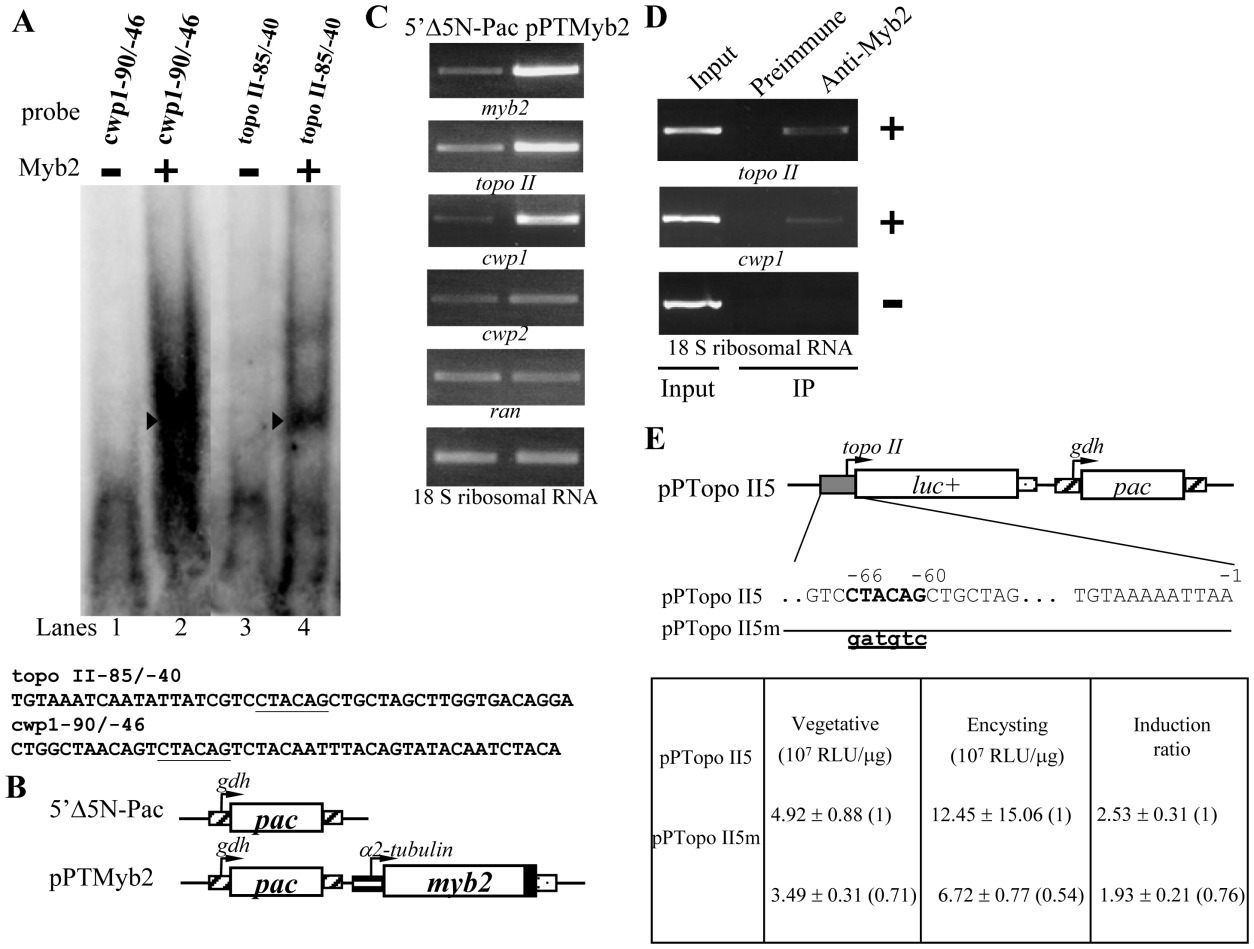
Activation of *topo II* gene expression in the Myb2 overexpressing cell line. (A) Detection of Myb2 binding site in *topo II* promoter. Electrophoretic mobility shift assays were performed using purified Myb2 and ^32^P-end-labeled cwp1-90/-46 and topo II-85/-40 probes as described. The Myb2 binding sequence is underlined. Components in the binding reaction mixtures are indicated above the lanes. The arrowheads indicate the shifted complex. (B) Diagrams of the 5′Δ5N-Pac and pPTMyb2 plasmids. The *pac* gene (open box) expression cassette is the same as in Fig. 1D. In construct pPTMyb2, the *myb2* gene is under the control of the constitutively expressed α2-tubulin promoter (stripled box) and the 3′-flanking region of the *ran* gene (dotted box). The filled box indicates the coding sequence of the AU1 epitope tag. (C) Overexpression of Myb2 increased the *topo II* gene expression. The 5′Δ5N-Pac and pPTMyb2 stable transfectants were cultured in encystation medium and then subjected to RT-PCR analysis. PCR was performed using primers specific for *myb2*, *topo II*, *cwp1*, *cwp2*, *ran*, and 18 S ribosomal RNA genes. As a control, similar mRNA levels of the 18 S ribosomal RNA gene were detected. (D) Recruitment of Myb2 to the *cwp1* and *topo II* promoters. The nontransfected WB cells were cultured in encystation medium for 24 h and then subjected to ChIP assays. Anti-Myb2 was used to assess binding of Myb2 to endogenous gene promoters. Preimmune serum was used as a negative control. Immunoprecipitated chromatin was analyzed by PCR using primers that amplify the 5′-flanking region of specific genes. At least three independent experiments were performed. Representative results are shown. Immunoprecipitated products of Myb2 yield more PCR products of *cwp1* and *topo II* promoters, indicating that Myb2 was bound to these promoters (+). The 18 S ribosomal RNA gene promoter was used as a negative control for our ChIP analysis (−). (E) Mutation analysis of the Myb2 binding site in the *topo II* promoter region. In the pPTopo II5 construct, a firefly luciferase gene (*luc+*, open box) is flanked by the 5′-flanking region of the *topo II* gene and 3′-flanking region of the *ran* gene (dotted box). The *pac* gene expression cassette is the same as in Fig. 1D. The Myb2 binding sequence is in boldface type. The mutated sequence in the construct pPTopo II5m is shown in underlined lowercase letters. After stable transfection with these constructs, luciferase activity was measured in vegetative cells and 24-h encysting cells as described under “Experimental Procedures”. Values are shown as means ± S.E. The induction ratio was obtained by dividing the activity in the encysting cells by the activity in the vegetative cells of each construct.

In the previous studies, we have identified a Myb2 transcription factor that is encystation-induced and is involved in coordinate up-regulation of *cwp1-3* genes and its own gene by binding to specific sequences [22], [24]. In previous studies, we have found that Myb2 can bind to the *cwp1* promoter [22]. To gain insight into the function of Topo II in cell differentiation, we tested the hypothesis that Myb2 can activate transcription of the endogenous *topo II* gene. We expressed Myb2 in *E. coli* and purified it to >95% homogeneity to test the DNA binding activity of Myb2 (data not shown). Electrophoretic mobility shift assays were performed with the purified Myb2 protein and double-stranded DNA sequences from the 5′-flanking region of *cwp1* and *topo II* genes. Incubation of a labeled double-stranded DNA probes cwp1-90/-46 with Myb2 resulted in the formation of retarded bands (Fig. 8A, lane 2), similar to our previous findings [22]. We also found that Myb2 also bound to the topo II-85/-40 probe (Fig. 8A, lane 4), which contains the Myb2 binding sequence, CTACAG [22].

In previous studies, we have found that the expression of the *cwp1* and *cwp2* genes was up-regulated by ∼3.6 and ∼3.8-fold in the Myb2 overexpressing cell line, respectively [24]. The endogenous *myb2* plus vector expressed *myb2* in the Myb2 overexpressing cell line increased by ∼2.7-fold [24]. We also found the mRNA levels of the *topo II* gene increased significantly in the Myb2 overexpressing cell line (Fig. 8B and C). We also found that the *topo II* gene is up-regulated by ∼2-fold in quantitative real-time PCR analysis (data not shown). As a control, the 18 S ribosomal RNA levels did not change in the Myb2 overexpressing cell line compared with the control cell line (Fig. 8C).The *ran* mRNA levels decreased in the Myb2 overexpressing cell line compared with the control cell line (Fig. 8C) [24].

We have used ChIP assays to confirm the binding of Myb2 to the *cwp1*, *cwp2*, and *myb2* gene promoters [24]. We further used ChIP assays to study association of Myb2 with the *topo II* promoter. As shown in Fig. 8D, Myb2 was associated with the *cwp1* and *topo II* promoters during encystation (Fig. 8D). However, Myb2 was not associated with the 18 S ribosomal RNA gene promoter, which has no Myb2 binding site (Fig. 8D) (data not shown).

We further investigated the ability of the Myb2 binding site to regulate the *topo II* promoter function by mutation analysis. The 5′-flanking region −300/−1 of the *topo II* gene was sufficient for up-regulation of the luciferase reporter gene during encystation (construct pPTopo II5, induction ratio ∼2.53) (Fig. 8E). Mutation from −66 to −60 of the *topo II* promoter, which spans a Myb2 binding site (in the region of the topo II-85/-40 probe), resulted in a significant decrease of luciferase activity to ∼71% and ∼54% of the wild-type value in vegetative and encysting cells, respectively, and a slight decrease in the induction to ∼1.93 (Fig. 8E). These results indicate that the Myb2 binding site in the *topo II* promoter function as a positive *cis*-acting element in both vegetative and encysting stages.

### A Topo II Inhibitor Has Anti-*Giardia* Effect

**Figure 9:**
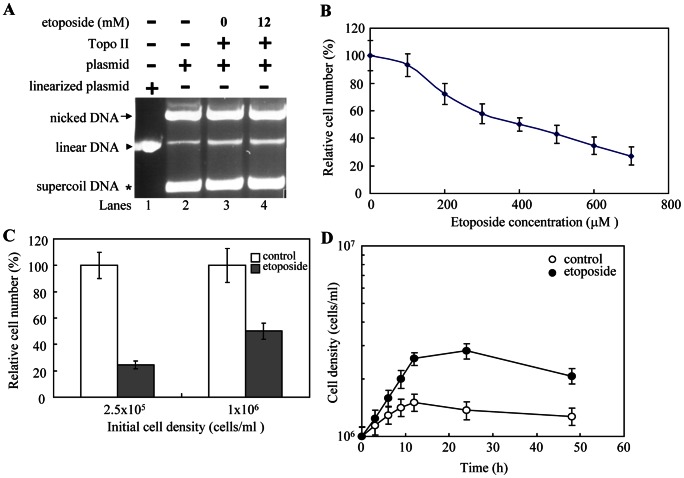
Anti-*Giardia* activity of etoposide. (A) Addition of etoposide increased DNA cleavage activity of Topo II. DNA cleavage assays are performed with purified recombinant Topo II and pUC119 plasmid in a buffer containing 5 mM magnesium II ion. Components in the reaction are indicated above the lanes. Typically, 2 ng Topo II was mixed with 300 ng plasmid DNA. Some reaction mixtures contain 12 mM etoposide, as indicated. Etoposide was dissolved in Me2SO. Adding Me2SO to the reaction mix increased the Topo II DNA cleavage activity (lane 3). Adding etoposide to the reaction mix increased the Topo II DNA cleavage activity (lane 4). Linearized plasmid is included as a size marker. (B) Dose effect of etoposide. The wild-type non-transfected WB cells were subcultured at an initial density of 1×10^6^ cells/ml in growth medium containing 0, 100, 200, 300, 400, 500, 600, or 700 µM etoposide for 24 h and then subjected to cell count. An equal volume ofMe2SO was added to cultures as a negative control. The sum of total cells is expressed as relative expression level over control. Values are shown as means ± S.E. of three independent experiments. (C) Effect of etoposide at different cell densities. The wild-type non-transfected WB cells were subcultured at an initial density of 2.5×10^5^ or 1×10^6^ cells/ml in growth medium containing 400 µM etoposide, or the same volume of Me2SO for 24 h and then subjected to cell count. The sum of total cells is expressed as relative expression level over control. Values are shown as means ± S.E. of three independent experiments. (D) Effect of etoposide on growth kinetics of *G*. *lamblia*. The wild-type non-transfected WB cells were subcultured at an initial density of 1×10^6^ cells/ml in growth medium containing 400 µM etoposide. An equal volume of Me2SO was added to cultures as a negative control. The cell density was monitored in triplicates over a 48 h time course by hematocytometer counting. Values are shown as means ± S.E. of three independent experiments.

Etoposide is a nonintercalating topoisomerase II inhibitor developed from a natural herb plant mayapple [70]. Etoposide binds and traps cleavage complex, resulting in cell apoptosis [51], [52]. We also performed DNA cleavage assays with etoposide. As shown in Fig. 9A, addition of etoposide slightly increased DNA cleavage activity of Topo II (Fig. 9A, lane 4). The results indicate that Topo II may function as a type II topoisomerase in *Giardia* and etoposide can trap cleavage complex of Topo II. We also found that etoposide has a significant anti-*Giardia* effect (Fig. 9B). Half-maximal inhibitory concentration (IC50) for etoposide was 400 µM (Fig. 9B). A higher anti-*Giardia* effect was found at a lower initial cell density (2.5×10^5^ cells/ml) (Fig. 9C). We also performed a time course study to understand the effect of etoposide on *Giardia* growth. Density of the etoposide treated cells was lower than that of control at different time points (Fig. 9D). Within the first 1 day, density of the etoposide-treated cells was decreased to 1.37×10^6^ cells/ml, while density of control cells was ∼2.71×10^6^ cells/ml (Fig. 9D).

**Figure 10:**
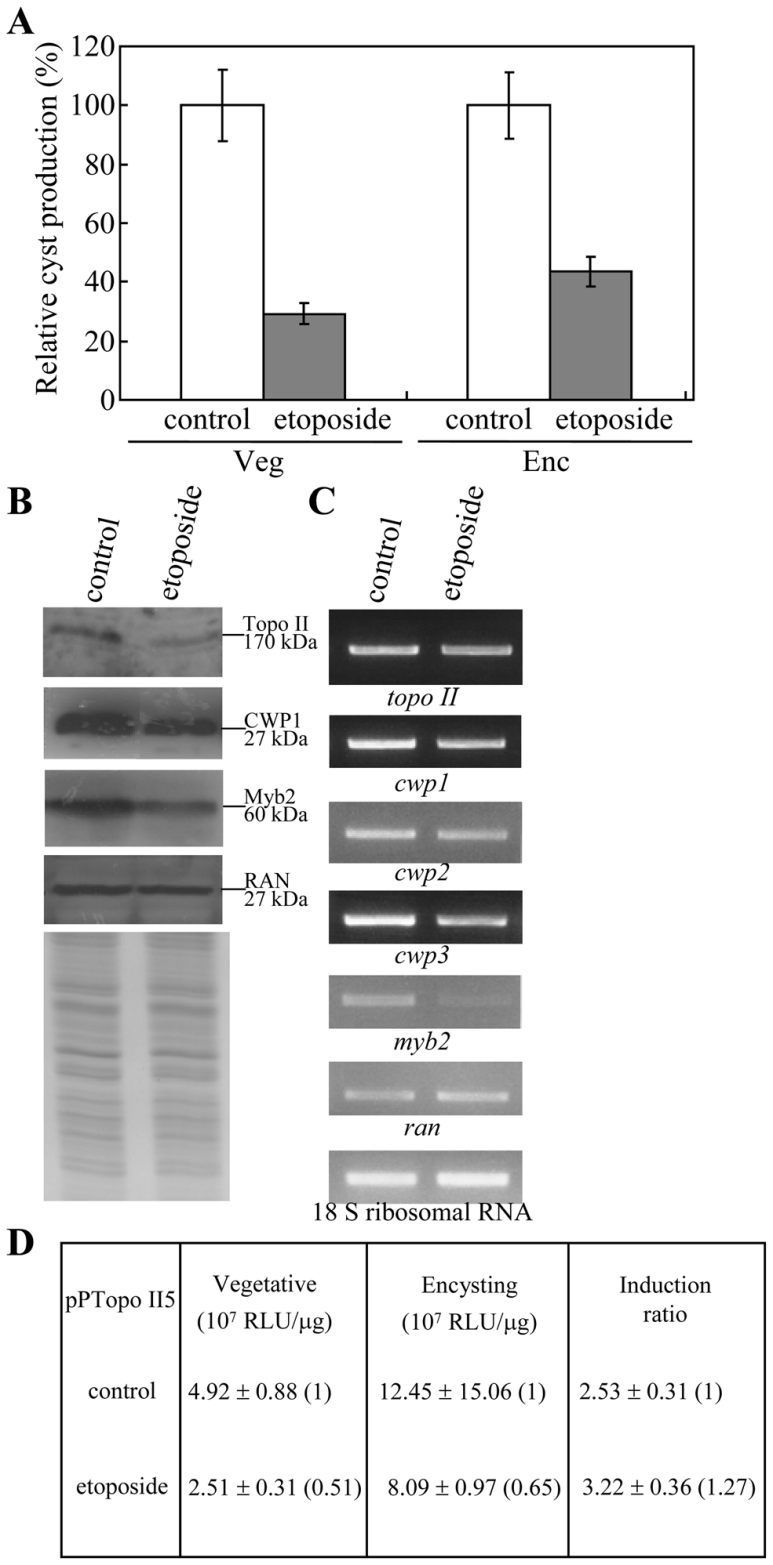
Inhibition of cyst formation by etoposide. (A) Addition of etoposide decreased the levels of cyst formation. The wild-type nontransfected WB cells were cultured in growth medium containing 400 µM etoposide, or the same volume of Me2SO for 24 h and then subjected to cyst count. The sum of total cysts is expressed as the relative expression level over control. Values are shown as mean ± S. E. of three independent experiments. (B) Addition of etoposide decreased the levels of Topo II, CWP1 and Myb2 proteins. The wild-type nontransfected WB cells were cultured in growth medium containing 400 µM etoposide, or the same volume of Me2SO for 24 h and then subjected to SDS-PAGE and Western blot. The blot was probed by anti-Topo II, anti-CWP1, anti-Myb2, and anti-RAN antibodies. Representative results are shown. Equal amounts of proteins loading were confirmed by SDS-PAGE and Coomassie Blue staining. (C) Addition of etoposide decreased the mRNA levels of the *topo II*, *cwp1*, *cwp2*, *cwp3*, and *myb2* genes. The wild-type nontransfected WB cells were cultured in growth medium containing 400 µM etoposide, or the same volume of Me2SO for 24 h and then subjected to RT-PCR analysis. PCR was performed using primers specific for the *topo II*, *cwp1*, *cwp2*, *cwp3*, *myb2*, *ran*, and 18 S ribosomal RNA. (D) Addition of etoposide decreased the *topo II* promoter activity. Data are presented as in Fig. 8E. The pPTopo II5 cells were cultured in growth medium or encystation medium containing 400 µM etoposide, or the same volume of Me2SO for 24 h and then subjected to luciferase activity. Values are shown as means ± S.E. The induction ratio was obtained by dividing the activity in the encysting cells by the activity in the vegetative cells of each construct.

Addition of etoposide also significantly decreased cyst formation during both vegetative growth and encystation (Fig. 10A). Interestingly, addition of etoposide also significantly decreased the levels of Topo II, CWP1, and Myb2 proteins (Fig. 10B). As a control, similar levels of intensity of the giardial RAN protein (∼27 kDa) were detected by anti-RAN antibody (Fig. 10B). RT-PCR and quantitative real time PCR analysis showed that addition of etoposide significantly decreased the mRNA levels of *topo II*, *cwp1*, *cwp2*, *cwp3*, and *myb2* genes by ∼0.61, ∼0.60, ∼0.51, ∼0.45, and ∼0.56-fold (*p*<0.05) (Fig. 10C and data not shown). Similar mRNA levels of the *ran* and 18 S ribosomal RNA genes were detected (Fig. 10C). Similar results were obtained during encystation (data not shown). Furthermore, addition of etoposide resulted in a significant decrease of luciferase activity to ∼51% and ∼65% of the wild-type value in vegetative and encysting cells, respectively, and a slight increase in the induction ratio to ∼3.22 (Fig. 10D). The results from the topoisomerase inhibitor etoposide suggest that Topo II may regulate *Giardia* differentiation into cysts through up-regulation of *cwp1-3* and *myb2* gene expression.

Oligonulceotide microarray assays confirmed the down-regulation of the *myb2* gene expression in the etoposide treated cells to ∼56% of the levels in the control cells (data not shown). Similar mRNA levels of the *ran* gene were detected (data not shown). We found that 56 and 48 genes were significantly up-regulated (>2-fold) and down-regulated (<1/2)(*p*<0.05) in the etoposide treated cells relative to the control cells, respectively (Table S3).

## Discussion

Type II topoisomerases are required for cell proliferation, tissue development, and cell cycle progression in higher eukaryotes [33], [34], [36], [39]. In this study, a type II topoisomerase has been identified and characterized in *G. lamblia* although divergent in sequence. This suggests that the type II topoisomerases may have evolved before divergence of *G. lamblia* from the main eukaryotic line of descent. Like Yeast or *Drosophila*, *Giardia* has only a single type II topoisomerase [71], [72], [73]. In kinetoplastid protozoa, such as *Trypanosoma brucei brucei*, the mitochondria complex kinetoplast DNA forms a catenated DNA network. To resolve topological problems, *Trypanosoma brucei brucei* has two type II topoisomerases in nucleus (Tbtop2 and Tbtop2β) and one in mitochondria (TbTOP2mt) [74].

The genes encoding key components of the giardial cyst wall, cyst wall proteins, are up-regulated during *Giardia* differentiation into dormant cysts [17], [18], [19]. During encystation, DNA is replicated and two nuclei divide without cytokinesis to form a cyst with four nuclei [1]. It has been shown that the gene of a key DNA synthesis enzyme, thymidine kinase, is up-regulated during encystation [27]. Because type II topoisomerase is important for relieving torsional stress during DNA replication [33], [34], we asked whether *Giardia* Topo II may help encystation. Our results show that the giardial Topo II localizes to the cell nuclei and an increase of the Topo II protein during encystation (Fig. 1C). We found that Topo II has ATPase activity and DNA cleavage activity (Figs. 4 and 5). We also found that Topo II can bind to specific sequences in the core AT-rich initiator region of the *cwp1*-*3* genes (Figs. 6 and 7). Interestingly, the constitutively overexpressed Topo II increased the levels of the CWP1 and Myb2 proteins and the *cwp1-3* and *myb2* mRNA and cyst formation (Fig. 3A–C). Oligonucleotide microarray assays confirmed the up-regulation of the *cwp1*, *cwp2*, and *myb2* gene expression in the Topo II overexpressing cell line (Fig. 3G and Table S2). Furthermore, addition of an inhibitor of type II topoisomerases, etoposide, inhibited cell growth and decreased the levels of the CWP1and Myb2 proteins, *cwp1-3* and *myb2* gene mRNA, and cyst formation (Figs. 9 and 10). The results suggest that Topo II may induce *cwp* gene expression and *Giardia* encystation.

The AT-rich initiator elements of the *ran*, *α2-tubulin*, and *cwp2* genes are positive *cis*-acting elements and that they are important for basal promoter activity and transcription start site selection [11], [12], [14], [15]. Previously, we have identified several transcription factors involved in the transactivation of the *cwp* genes [15], [22], [23], [24], [25], [27], [28]. ARID1, Pax1, and Pax2 can bind to the AT-rich Inr elements of the *cwp* promoters [15], [26], [28]. Myb2, GARP1, WRKY, and E2F1 can bind to the proximal upstream regions of the *cwp* promoters and their binding sequences are positive *cis*-acting elements [22], [23], [25], [27]. It has been reported that topoisomerase II enhances transcription by binding to nucleosome-free promoters and recruiting RNA polymerase II in yeast [40]. In this study, we also found that Topo II can also bind to the AT-rich initiator elements of the *cwp* and *myb2* promoters *in vitro* (Fig. 7C). An etoposide-mediated topoisomerase immunoprecipitation assays also confirmed the association of Topo II with its own promoter and the *cwp* and *myb2* promoters *in vivo* (Fig. 7E). There may be an interaction of the Topo II and transcription factors binding to the proximal upstream regions and the AT-rich Inr elements. This interaction may be required for promoter activity and accurate transcription start site selection. Topo II can bind AT-rich initiator elements of both the constitutive *ran* gene and encystation-induced *cwp* and *myb2* genes (Fig. 7C and 7E). However, overexpressed Topo II induced the expression of the *cwp* promoters but did not induce the expression of the *ran* promoter (Fig. 3). This could be due to a cooperation of encystation-specific transcription factors to transactivate the encystation-induced *cwp* genes. Similar results were found in overexpression of encystation-induced transcription factors [24], [25], [26], [27], [28].

Although divergent from human topoisomerase IIα/β proteins, the full-length *Giardia* Topo II has ATPase activity, decatenation activity, cleavage activity, and DNA binding activity (Figs. 4–6). This indicates that function of the *Giardia* Topo II may have been conserved in evolution. In the ATPase assays, the specific activity of the Giardia Topo II is 108.30 µmol PO4·min−1·mg−1, which is higher than the specific activity of the human topoisomerase IIα (0.24 µmol PO4·min−1·mg−1) [75]. The ATPase activity of the human topoisomerase IIα can be stimulated by the presence of DNA [76]. We also found that addition of DNA resulted in an increase of ATPase activity of the full-length Topo II (Fig. 5A). The Km value decreased significantly with the addition of DNA and the Vmax value increased significantly with the addition of DNA (Fig. 5B). Deletion of the N terminal region (Topo IIC) that contains the ATPase domain resulted in a loss of ATPase activity (Fig. 5A). In contrast, deletion of the C terminal region that contains the cleavage domain (Topo IIN) did not affect the ATPase activity but resulted in a loss of DNA dependent ATPase activity, possibly due to the lack of interdomain communication (Fig. 5A). Similar phenomena have been found in human Topo IIα [42]. Coumermycin A1 can inhibit the ATPase activity of the bacterial DNA gyrase but not that of the eukaryotic type II topoisomerases [77]. We found that addition of 400 µM of coumermycin A1 did not affect the ATPase activity of the full-length Topo II (data not shown), suggesting that the *Giardia* Topo II functions more like the eukaryotic type II topoisomerases.

It has been shown that magnesium is a requirement for activity of the human type II topoisomerases [78]. Four residues of human topoisomerase IIα (topoisomerase IIβ) are involved in magnesium coordination and important for catalytic activity, including Glu 461, Asp 541, Asp 543, and Asp 545 (Glu 477, Asp 557, Asp 559, and Asp 561) [78]. These residues are conserved in *Giardia* Topo II (Fig. S1). In addition, magnesium II ion is required for DNA cleavage activity of Topo II (Fig. 4B), suggesting that *Giardia* Topo II functions like a type II topoisomerase. The cleavage domain (Topo IV domain, residues residues 755 to 1322) of *Giardia* Topo II is near the C terminus (Fig. 4A). Deletion of the C terminal region resulted in a loss of cleavage activity and DNA binding activity (Topo IIN) (Figs. 4C and 6), but deletion of the N terminal region did not affect the cleavage activity and DNA binding activity (Topo IIC) (Figs. 4D and 6). Three mutants based on the Topo IIC backbone were created and tested. Deletion of C terminal 153 amino acids (residues 1339–1491, Topo IICm2) did not affect the cleavage activity but slightly decreased DNA binding activity (Figs. 4E and 6). However, deletion of the C-terminal region containing the Topo IV domain (residues 858–1491, Topo IICm3) resulted in a significant decrease of cleavage activity and DNA binding activity (Figs. 4E and 6). We also found a mutation of the catalytic important Tyr 847 (Topo IICm1) resulted in a significant loss of cleavage activity (Fig. 4E)(see below). However, mutation of this important Tyr did not affect its DNA binding activity (Fig. 6, lane 6).

A specific Tyr of the type II topoisomerases forms a covalent complex with DNA to create a transient double stranded DNA break during cycles of DNA breakage and religation [33], [34]. A mutation of active site Tyr of Topoisomerase IV with histidine resulted in a loss of transesterification activity [79]. We also performed mutation analysis to understand whether Tyr 847 of Topo II, which corresponds to Tyr 805 of the human topoisomerase IIα, is also important for its activity (Fig. S1). We found that mutation of the Tyr 847 to His resulted in loss of nuclear localization in both vegetative and encysting cells (Fig. 2B–G, Topo IIm1), suggesting that the Tyr 847 residue may play an important role in the exclusively nuclear localization. Mutation of this important Tyr also resulted in a significant decrease of the levels of CWP1 protein, cyst formation, *cwp1-3* and *myb2* mRNA, and DNA cleavage activity (Topo IICm1) (Figs. 3 and 4). Similar results were obtained when the Tyr was mutated to Trp (data not shown). We also found that deletion of the C-terminal 153 amino acids (residues 1339–1491, pPTopo IIm2, Fig. 2H–M) resulted in loss of nuclear localization. Nuclear localization signals typically are regions rich with basic amino acids. Several typical nuclear localization signals were predicted in Topo II using the PSORT program (http://psort.nibb.ac.jp/), including PKTKRTK at 295, RKVLYACFKRNLKTKLK at 769, PSRKHRI at 1200, PKPKKEH at 1395, KPKK at 1396, PTEPKRK at 1424, PKRKRPA at 1427, PKRK at 1427, KRKR at 1428, RKRP at 1429. Deletion of the C-terminal 153 amino acids (residues 1338 to 1491) (pPTopo IIm2) (Fig. 2A) resulted in loss of nuclear localization in both vegetative and encysting cells (Fig. 2H–M), suggesting that this region may play an important role in the exclusively nuclear localization. Deletion of the C-terminal 153 amino acids resulted in a significantly decrease of the levels of CWP1 protein, cyst formation, and *cwp1-3* and *myb2* mRNA (Topo IIm2) (Figs. 3E and S5A), but the effect is lower than the Topo IIm1 and Topo IIm3. Interestingly, DNA cleavage activity was not affected in this mutant (Topo IICm2) (Fig. 4), suggesting a correlation of DNA cleavage activity and *in vivo* function. As discussed above, Topo IIm2 still has some ability to induce *cwp* and *myb2* gene expression, even though it is not localized to the nucleus (Figs. 2B, 3E and S5A). Deletion of the C-terminal region containing the Topo IV domain (residues 858–1491, pPTopo IIm3, Fig. 2N–S) resulted in loss of nuclear localization. Deletion of this region resulted in a significant decrease of the levels of CWP1 protein, cyst formation, *cwp1-3* and *myb2* mRNA, and DNA cleavage activity (Topo IICm3) (Figs. 3 and 4). The results suggest that Topo II may induce the expression of encystation-induced *cwp1-3* and *myb2* genes *in vivo* through its cleavage activity.

Topoisomerases may affect chromosome dynamics and thereby activate gene expression [80]. Inactivation of topoisomerases may reduce rRNA and mRNA synthesis [81], [82]. Mammalian Topoisomerase IIβ has an important role in inducing neuronal development [38], [39]. It is interesting that it can induce transcription of specific genes required for neuronal development and that location of its target genes is closed to AT-rich intergenic regions [38], [39]. Our results indicate that the AT-rich initiator sequence may be important for binding of *Giardia* Topo II (Fig. 7C). Further studies also indicate that Topo II can bind to poly(A) sequence with a T, TT, or TC insertion (Fig. 7C). We also found that Topo II can not bind to the 18 S ribosomal RNA gene promoter that is GC rich and does not contain the AT-rich initiator (18S-30/-1 and 18S-60/-31) (Fig. 7B). In addition, we found a decrease of DNA binding activity of Topo IICm3 (Fig. 6, lane 8), indicating that the cleavage domain is important for full DNA binding activity of Topo II. Our results suggest that *Giardia* Topo II may bind and regulate *cwp* gene promoters to induce *Giardia* encystation. The variably regulated *vsp* gene expression is important for *Giardia* pathogenicity [83]. We also found Topo II may bind to a *vsp* gene promoter which is not very AT-rich (Fig. S6). This *vsp* gene was up-regulated by Topo II overexpression (Table S2; orf number 137620). This could be due to a cooperation of *vsp*-specific transcription factors to transactivate the *vsp* gene. Further studies are required to characterize and evaluate the potential transcription mechanism of positive and negative regulation of *vsp* genes in *Giardia*.

It has been shown that etoposide is a potent inhibitor of the human topoisomerase IIα/β [51], [52]. Etoposide can trap the cleavage complex and prevent religation of DNA, resulting in double stranded break, extensive DNA fragmentation, and cell apoptosis [51], [52]. We found that addition of etoposide increased DNA cleavage activity of Topo II (Fig. 9A). We also found that addition of etoposide significantly decreased cyst formation and cell growth (Figs. 9 and 10). Addition of etoposide decreased the levels of Topo II, CWP1, and Myb2 proteins and levels of *cwp1-3* and *myb2* mRNA (Fig. 10B and C). Oligonucleotide microarray assays confirmed the down-regulation of the *myb2* gene expression in the etoposide treated cells to ∼56% of the levels in the control cells (data not shown). We also found that addition of etoposide decreased the *topo II* promoter activity (Fig. 10D). The half maximal inhibitory concentration (IC50) of etoposide used in the assays is 400 µM, and this concentration may kill many human cell lines (the half maximal inhibitory concentration for human breast cancer MCF-7 Cells is ∼10 µM) [84]. Our results suggest that etoposide can inhibit topoisomerase function, thereby decreasing *cwp* gene expression. Further studies are required to find more suitable topoisomerase inhibitors to inhibit *Giardia* cyst formation and growth but not to harm human cells. Metronidazole has been used often in the treatment of *Giardia* infection with an IC50 of 2.1 µM [55], [85]. Our results suggest that etoposide is less effective than the standard drug metronidazole. This could be due to the variability of the C terminal regions and overall sequences of topoisomerases II from *Giardia* and higher eukaryotes and the region are helpful for designing therapeutic drugs [43], [44], [45], [46].

In previous studies, we have identified a Myb2 transcription factor that is encystation induced and is involved in coordinate up-regulation of key encystation-induced genes, *cwp1-3* and *myb2* itself [22], [24]. In this study, we found that the Myb2 binding site is present in the proximal 5′-flanking region of the *topo II* gene and Myb2 can bind to the *topo II* promoter (Fig. 8A). Interestingly, overexpression of Myb2 induced the expression of *topo II* gene (Fig. 8C). ChIP assays confirmed the association of Myb2 with the *topo II* promoter (Fig. 8D). Mutation analysis of the *topo II* promoter has provide evidence for involvement of Myb2 binding site during vegetative growth and encystation (Fig. 8E). The Myb2 binding site is more important during encystation, because the activity of the *topo II* promoter with a mutation of Myb2 binding site decreased more during encystation (Fig. 8E). The results suggest that Myb2 may play a role in induction of *topo II* expression. Similarly, c-Myb has also been found to induce human topoisomerase IIα gene expression [86], [87]. Interestingly, we also found that *Giardia* Topo II can induce the expression of *myb2* gene (Fig. 3A and 3B). The results suggest a positive regulation cycle between Topo II and Myb2. In addition, addition of etoposide resulted in a decrease of *topo II* and *myb2* gene expression (Fig. 10B and 10C) and etoposide can decrease the promoter activity of *topo II* gene (Fig. 10D). Therefore, it is possible that etoposide may decrease the *topo II* gene expression through down-regulation of Myb2 in *Giardia*.

We found that 95 and 20 genes were significantly up-regulated (>2-fold) and down-regulated (<1/2)(*p*<0.05) in the Topo II overexpressing cell line relative to the vector control using oligonucleotide microarray assays (Table S2). We also found that 56 and 48 genes were significantly up-regulated (>2-fold) and down-regulated (<1/2)(*p*<0.05) in the etoposide treated cells relative to the control cells, respectively (Table S3). Interestingly, two multidrug resistance-associated protein 1 (open reading frames 41118 and 115052) were up-regulated by etoposide treatment (Table S3). It has been shown that multidrug resistance associated proteins are up-regulated and associated with drug resistance during treatment of anticancer drugs such as doxorubicin and etoposide [88]. Our results suggest that multidrug resistance-associated proteins may also play a role in resistance to etoposide treatment in *Giardia*. Seven genes listed in Table S2, including six variant-specific surface proteins and one high cysteine membrane protein group 1 (open reading frames 41476, 103992, 9276, 112113, 115797, 115796, and 25816), were both up-regulated by Topo II overexpression and etoposide treatment (Table S3). Only one gene listed in Table S2, variant-specific surface protein (open reading frame 115047), was down-regulated by Topo II overexpression and up-regulated by etoposide treatment (Table S3).

Regulation of chromatin reorganization by Top2β plays a role in gene expression and determines neuronal cell differentiation [89]. Interestingly, we also showed that Topo II can induce the expression of the *cwp* genes that are involved in differentiation in the primitive protozoan *G. lamblia*., suggesting that giardial Topo II may be functionally conserved, involved in regulation of gene expression and cell differentiation. Our study provides evidence for the important role of Topo II in the differentiation of *G. lamblia* trophozoites into cysts, leading to greater understanding of the evolution of eukaryotic topoisomerases during cell differentiation.

## Supporting Information

Figure S1 Alignment of the full-length sequences of the Topo II proteins.
(PDF)

Figure S2 Alignment of the Topo IV domains of the Topo II proteins.
(PDF)

Figure S3 Phylogenetic analysis of Topo II proteins.
(PDF)

Figure S4 Induction of *cwp1-3* and *myb2* gene expression in the Topo II overexpressing cell line during encystation.
(PDF)

Figure S5 Analysis of Topo II function.
(PDF)

Figure S6 Binding of Topo IIC to *vsp* promoter.
(PDF)

Table S1 Oligonucleotides used in this study.
(PDF)

Table S2 Genes up or down regulated by Topo II overexpression
(PDF)

Table S3 Genes up or down regulated by etoposide treatment
(PDF)
