# Correction: DNA Topoisomerase II Is Involved in Regulation of Cyst Wall Protein Genes and Differentiation in *Giardia lamblia*

**DOI:** 10.1371/journal.pntd.0005326

**Published:** 2017-01-27

**Authors:** Bo-Chi Lin, Li-Hsin Su, Shih-Che Weng, Yu-Jiao Pan, Nei-Li Chan, Tsai-Kun Li, Hsin-Chih Wang, Chin-Hung Sun

Fig 1, Fig 3 and Fig 9 are incorrect. Please see the corrected versions here.

**Fig 1:**
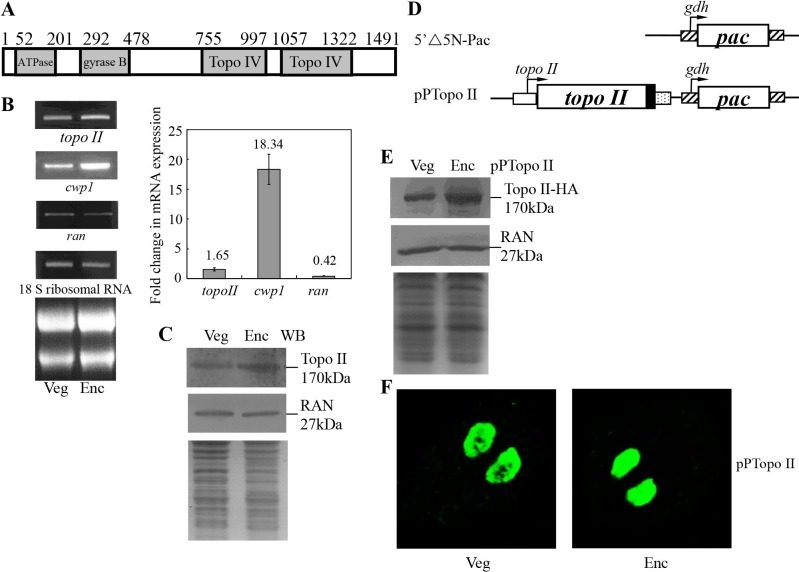
Analysis of topo II gene expression. (A) Schematic representation of the Giardia Topo II protein. The gray boxes indicate the ATPase, gyrase B, and Topo IV domains, as predicted by pfam (http://pfam.sanger.ac.uk/) [65]. (B) RT-PCR and quantitative real-time PCR analysis of topo II gene expression. RNA samples were prepared from G. lamblia wild type nontransfected WB cells cultured in growth (Veg, vegetative growth) or encystation medium and harvested at 24 h (Enc, encystation). RT-PCR was performed using primers specific for topo II, cwp1, ran, and 18 S ribosomal RNA genes. Ribosomal RNA quality and loading controls are shown in the bottom panel. Representative results are shown on the left. Real-time PCR was preformed using primers specific for topo II, cwp1, ran, and 18 S ribosomal RNA genes. Transcript levels were normalized to 18 S ribosomal RNA levels. -Fold changes in mRNAexpression are shown as the ratio of transcript levels in encysting cells relative to vegetative cells. Results are expressed as the means ± S.E. (error bars) of at least three separate experiments (right). (C) Topo II protein levels in different stages. The wild type nontransfected WB cells were cultured in growth (Veg, vegetative growth) or encystation medium for 24 h (Enc, encystation) and then subjected to SDS-PAGE and Western blot. The blot was probed by anti-Topo II and anti-RAN antibody. Representative results are shown. Equal amounts of protein loading were confirmed by SDS-PAGE and Coomassie Blue staining. (D) Diagrams of the 5′Δ5N-Pac and pPTopo II plasmid. The pac gene (open box) is under the control of the 5′- and 3′-flanking regions of the gdh gene (striated box). In construct pPTopo II, the topo II gene is under the control of its own 5′-flanking region (open box) and the 3′-flanking region of the ran gene (dotted box). The filled black box indicates the coding sequence of the HA epitope tag. (E) Topo II protein levels increased during encystation. The pPTopo II stable transfectants were cultured in growth (Veg, vegetative growth) or encystation medium for 24 h (Enc, encystation) and then subjected to SDS-PAGE and Western blot. HA-tagged Topo II protein was detected in the pPTopo II stable transfectants using an anti-HA antibody by Western blot analysis. Equal amounts of protein loading were confirmed by anti-Ran Western blot and SDS-PAGE with Coomassie Blue staining. (F) Nuclear localization of Topo II. The pPTopo II stable transfectants were cultured in growth (Veg, vegetative growth, left panel) or encystation medium for 24 h (Enc, encystation, right panel) and then subjected to immunofluorescence analysis using anti-HA antibody for detection. The product of pPTopo II localizes to the nuclei in both vegetative and encysting trophozoites.Fig 3. Effect of humidity and temperature on the frequency of dengue cases. Solid and dotted lines represents humidity and temperature respectively, whereas vertical bars represents frequency of dengue cases during the months of July to December.

**Fig 3:**
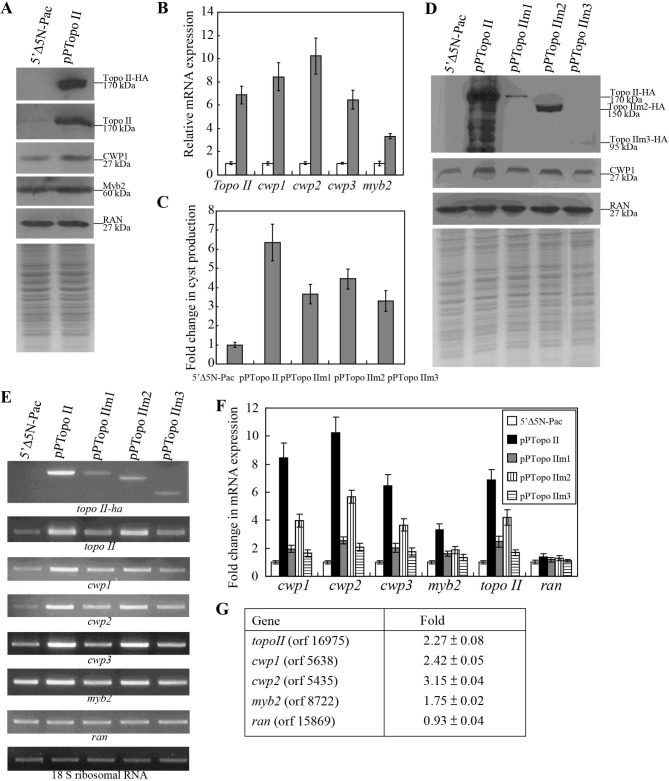
Induction of cwp1-3 and myb2 gene expression in the Topo II overexpressing cell line. (A) Overexpression of Topo II increased the levels of CWP1 protein. The 5′Δ5N-Pac and pPTopo II stable transfectants were cultured in growth medium and then subjected to SDS-PAGE and Western blot. The blot was probed by anti-HA, anti-Topo II, anti-CWP1, anti-Myb2, and anti-Ran antibodies. Equal amounts of protein loading were confirmed by SDS-PAGE and Coomassie Blue staining. Representative results are shown. (B) Quantitative real-time PCR analysis of gene expression in the Topo II -overexpressing cell line. The 5′▵5N-Pac and pPTopo II stable transfectants were cultured in growth medium and then subjected to quantitative real-time PCR analysis. Real-time PCR was preformed using primers specific for topo II, cwp1, cwp2, cwp3, myb2, ran, and 18 S ribosomal RNA genes. Similar mRNA levels of the ran and 18 S ribosomal RNA genes for these samples were detected (data not shown). Transcript levels were normalized to 18 S ribosomal RNA levels. Fold changes in mRNA expression are shown as the ratio of transcript levels in the pPTopo II cell line relative to the 5′▵5N-Pac cell line. Results are expressed as the means ± S. E. of at least three separate experiments. (C) Cyst count. The 5′Δ5N-Pac, pPTopo II, pPTopo IIm1, pPTopo IIm2, and pPTopo IIm3 stable transfectants were cultured in growth medium and then subjected to cyst count as described under “Experimental Procedures”. The sum of total cysts is expressed as relative expression level over control. Values are shown as means ± S. E. (D) Analysis of Topo II mutants. The 5′Δ5N-Pac, pPTopo II, pPTopo IIm1, pPTopo IIm2, and pPTopo IIm3 stable transfectants were cultured in growth medium and then subjected to SDS-PAGE and Western blot. The blot was probed by anti-HA, anti-CWP1, and anti Ran antibodies. Equal amounts of protein loading were confirmed by SDS-PAGE and Coomassie Blue staining. Representative results are shown. (E) RT-PCR analysis of gene expression in the Topo II- and Topo II mutants- overexpressing cell lines. The 5′Δ5N-Pac, pPTopo II, pPTopo IIm1, pPTopo IIm2, and pPTopo IIm3 stable transfectants were cultured in growth medium and then subjected to RT-PCR analysis. PCR was performed using primers specific for topo II-ha, topo II, cwp1, cwp2, cwp3, myb2, ran, and 18 S ribosomal RNA genes. (F) Quantitative real-time PCR analysis of gene expression in the Topo II and Topo IIm1-3 overexpressing cell lines. Real-time PCR was performed using primers specific for topo II, cwp1, cwp2, cwp3, myb2, ran, and 18 S ribosomal RNA genes. Similar mRNA levels of the 18 S ribosomal RNA genes for these samples were detected. Transcript levels were normalized to 18 S ribosomal RNA levels. Fold changes in mRNA expression are shown as the ratio of transcript levels in the pPTopo II or pPTopo IIm1-3 cell line relative to the 5′Δ5N-Pac cell line. Results are expressed as the means ± standard error of at least three separate experiments. (G) Microarray analysis. Microarray data were obtained from the 5′Δ5N-Pac and pPTopo II cell lines during vegetative growth. Fold-changes are shown as the ratio of transcript levels in the pPTopo II cell line relative to the 5′Δ5N-Pac cell line. Results are expressed as the mean ± S. E. of at least three experiments.

**Fig 9:**
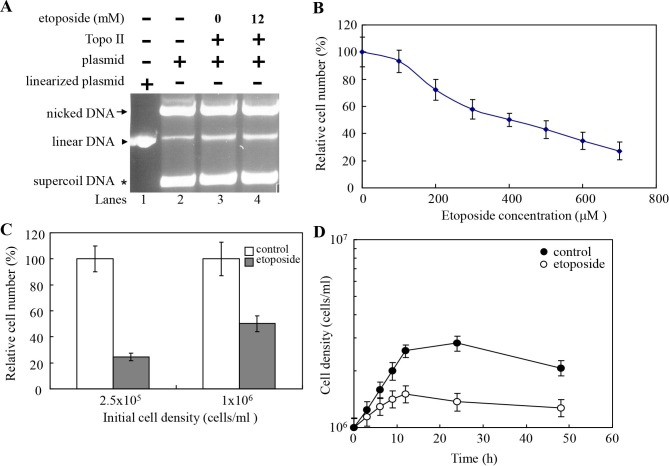
Anti-Giardia activity of etoposide. (A) Addition of etoposide increased DNA cleavage activity of Topo II. DNA cleavage assays are performed with purified recombinant Topo II and pUC119 plasmid in a buffer containing 5 mM magnesium II ion. Components in the reaction are indicated above the lanes. Typically, 2 ng Topo II was mixed with 300 ng plasmid DNA. Some reaction mixtures contain 12 mM etoposide, as indicated. Etoposide was dissolved in Me2SO. Adding Me2SO to the reaction mix increased the Topo II DNA cleavage activity (lane 3). Adding etoposide to the reaction mix increased the Topo II DNA cleavage activity (lane 4). Linearized plasmid is included as a size marker. (B) Dose effect of etoposide. The wild-type non-transfected WB cells were subcultured at an initial density of 1×106 cells/ml in growth medium containing 0, 100, 200, 300, 400, 500, 600, or 700 μM etoposide for 24 h and then subjected to cell count. An equal volume ofMe2SO was added to cultures as a negative control. The sum of total cells is expressed as relative expression level over control. Values are shown as means ± S.E. of three independent experiments. (C) Effect of etoposide at different cell densities. The wild-type non-transfected WB cells were subcultured at an initial density of 2.5×105 or 1×106 cells/ml in growth medium containing 400 μM etoposide, or the same volume of Me2SO for 24 h and then subjected to cell count. The sum of total cells is expressed as relative expression level over control. Values are shown as means ± S.E. of three independent experiments. (D) Effect of etoposide on growth kinetics of G. lamblia. The wild-type non-transfected WB cells were subcultured at an initial density of 1×106 cells/ml in growth medium containing 400 μM etoposide. An equal volume of Me2SO was added to cultures as a negative control. The cell density was monitored in triplicates over a 48 h time course by hematocytometer counting. Values are shown as means ± S.E. of three independent experiments.
