# Supplementary material for: DNA Topoisomerase II Is Involved in Regulation of Cyst Wall Protein Genes and Differentiation in Giardia lamblia
Source: PLoS Negl Trop Dis. 2013 May 16;7(5):e2218. doi: 10.1371/journal.pntd.0002218 (PMC3656124; doi:10.1371/journal.pntd.0002218)
Supplement: Figure S1 — Alignment of the full-length sequences of the Topo II proteins. (PDF) [file pntd.0002218.s001.pdf]

# Figure S1

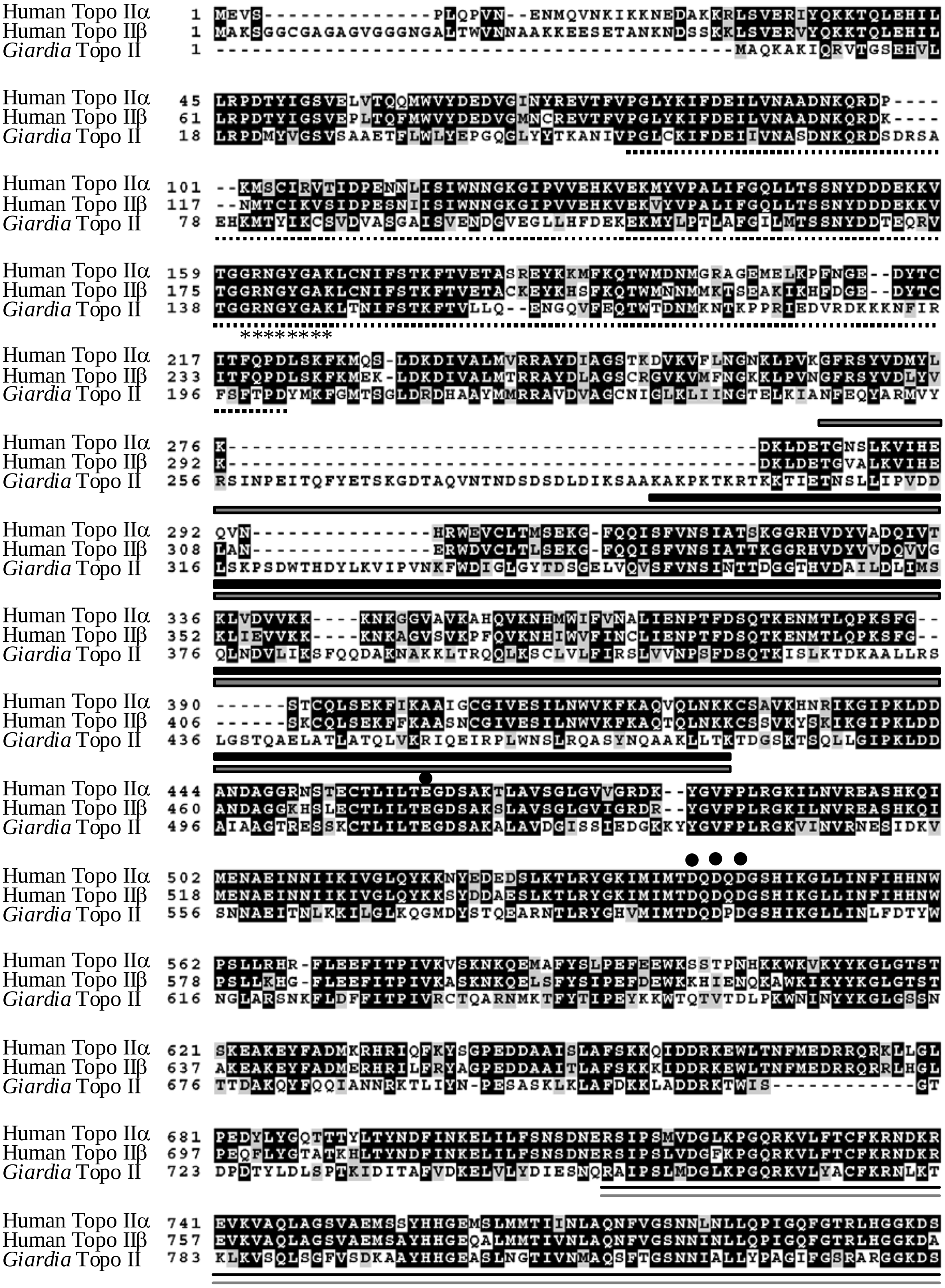

Human Topo IIα 1 MEVS--------------PLQPVN--ENMQVNKIKKNEDAKKRLSVERIYQKKTQLEHIL
Human Topo IIβ 1 MAKSGGCGAGAGVGGGNGALTWVNNAAKKEESETANKNDSSKKLSVERVYQKKTQLEHIL
Giardia Topo II 1 -------------------------------------------MAQKAKIQRVTGSEHVL
Human Topo IIα 45 LRPDTYIGSVELVTQQMWVYDEDVGINYREVTFVPGLYKIFDEILVNAADNKQRDP----
Human Topo IIβ 61 LRPDTYIGSVEPLTQFMWVYDEDVGMNCREVTFVPGLYKIFDEILVNAADNKQRDK----
Giardia Topo II 18 LRPDMYVGSVSAAETFLWLYEPGQGLYYTKANIVPGLCKIFDEIIVNASDNKQRDSDRSA
Human Topo IIα 101 --KMSCIRVTIDPENNLISIWNNGKGIPVVEHKVEKMYVPALIFGQLLTSSNYDDDEKKV
Human Topo IIβ 117 --NMTCIKVSIDPESNIISIWNNGKGIPVVEHKVEKVYVPALIFGQLLTSSNYDDDEKKV
Giardia Topo II 78 EHKMTYIKCSVDVASGAISVENDGVEGLLHFDEKEKMYLPTLAFGILMTSSNYDDTEQRV
Human Topo IIα 159 TGGRNGYGAKLCNIFSTKFTVETASREYKKMFKQTWMDNMGRAGEMELKPFNGE--DYTC
Human Topo IIβ 175 TGGRNGYGAKLCNIFSTKFTVETACKEYKHSFKQTWMNNMMKTSEAKIKHFDGE--DYTC
Giardia Topo II 138 TGGRNGYGAKLTNIFSTKFTVLLQ--ENGQVFEQTWTDNMKNTKPPRIEDVRDKKKNFIR
********
Human Topo IIα 217 ITFQPDLSKFKMQS-LDKDIVALMVRRAYDIAGSTKDVKVFLNGNKLPVKGFRSYVDMYL
Human Topo IIβ 233 ITFQPDLSKFKMEK-LDKDIVALMTRRAYDLAGSCRGVKVMFNGKKLPVNGFRSYVDLYV
Giardia Topo II 196 FSFTPDYMKFGMTSGLDRDHAAYMMRRAVDVAGCNIGLKLIINGTELKIANFEQYARMVY
Human Topo IIα 276 K--------------------------------------------DKLDETGNSLKVIHE
Human Topo IIβ 292 K--------------------------------------------DKLDETGVALKVIHE
Giardia Topo II 256 RSINPEITQFYETSKGDTAQVNTNDSDSDLDIKSAAKAKPKTKRTKKTIETNSLLIPVDD
Human Topo IIα 292 QVN---------------HRWEVCLTMSEKG-FQQISFVNSIATSKGGRHVDYVADQIVT
Human Topo IIβ 308 LAN---------------ERWDVCLTLSEKG-FQQISFVNSIATTKGGRHVDYVVDQVVG
Giardia Topo II 316 LSKPSDWTHDYLKVIPVNKFWDIGLGYTDSGELVQVSFVNSINTTDGGTHVDAILDLIMS
Human Topo IIα 336 KLVDVVKK----KNKGGVAVKAHQVKNHMWIFVNALIENPTFDSQTKENMTLQPKSFG--
Human Topo IIβ 352 KLIEVVKK----KNKAGVSVKPFQVKNHIWVFINCLIENPTFDSQTKENMTLQPKSFG--
Giardia Topo II 376 QLNDVLIKSFQQDAKNAKKLTRQQLKSCLVLFIRSLVVNPSFDSQTKISLKTDKAALLRS
Human Topo IIα 390 ------STCQLSEKFIKAAIGCGIVESILNWVKFKAQVQLNKKCSAVKHNRIKGIPKLDD
Human Topo IIβ 406 ------SKCQLSEKFFKAASNCGIVESILNWVKFKAQTQLNKKCSSVKYSKIKGIPKLDD
Giardia Topo II 436 LGSTQAELATLATQLVKRIQEIRPLWNSLRQASYNQAAKLLTKTDGSKTSQLLGIPKLDD
Human Topo IIα 444 ANDAGGRNSTECTLILTEGDSAKTLAVSGLGVVGRDK--YGVFPLRGKILNVREASHKQI
Human Topo IIβ 460 ANDAGGKHSLECTLILTEGDSAKSLAVSGLGVIGRDR--YGVFPLRGKILNVREASHKQI
Giardia Topo II 496 AIAAGTRESSKCTLILTEGDSAKALAVDGISSIEDGKKYYGVFPLRGKVINVRNESIDKV
Human Topo IIα 502 MENAEINNIIKIVGLQYKKNYEDEDSLKTLRYGKIMIMTDQDQDGSHIKGLLINFIHHNW
Human Topo IIβ 518 MENAEINNIIKIVGLQYKKSYDDAESLKTLRYGKIMIMTDQDQDGSHIKGLLINFIHHNW
Giardia Topo II 556 SNNAEITNLKKILGLKQGMDYSTQEARNTLRYGHVMIMTDQDPDGSHIKGLLINLFDTYW
Human Topo IIα 562 PSLLRHR-FLEEFITPIVKVSKNKQEMAFYSLPEFEEWKSSTPNHKKWKVKYYKGLGTST
Human Topo IIβ 578 PSLLKHG-FLEEFITPIVKASKNKQELSFYSIPEFDEWKKHIENQKAWKIKYYKGLGTST
Giardia Topo II 616 NGLARSNKFLDFFITPIVRCTQARNMKTFYTIPEYKKWTQTVTDLPKWNINYYKGLGSSN
Human Topo IIα 621 SKEAKEYFADMKRHRIQFKYSGPEDDAAISLAFSKKQIDDRKEWLTNFMEDRRQRKLLGL
Human Topo IIβ 637 AKEAKEYFADMERHRILFRYAGPEDDAAITLAFSKKKIDDRKEWLTNFMEDRRQRRLHGL
Giardia Topo II 676 TTDAKQYFQQIANNRKTLIYN-PESASKLKLAFDKKLADDRKTWIS------------GT
Human Topo IIα 681 PEDYLYGQTTTYLTYNDFINKELILFSNSDNERSIPSMVDGLKPGQRKVLFTCFKRNDKR
Human Topo IIβ 697 PEQFLYGTATKHLTYNDFINKELILFSNSDNERSIPSLVDGFKPGQRKVLFTCFKRNDKR
Giardia Topo II 723 DPDTYLDLSPTKIDITAFVDKELVLYDIESNQRAIPSLMDGLKPGQRKVLYACFKRNLKT
Human Topo IIα 741 EVKVAQLAGSVAEMSSYHHGEMSLMMTIINLAQNFVGSNNLNLLQPIGQFGTRLHGGKDS
Human Topo IIβ 757 EVKVAQLAGSVAEMSAYHHGEQALMMTIVNLAQNFVGSNNINLLQPIGQFGTRLHGGKDA
Giardia Topo II 783 KLKVSQLSGFVSDKAAYHHGEASLNGTIVNMAQSFTGSNNIALLYPAGIFGSRARGGKDS

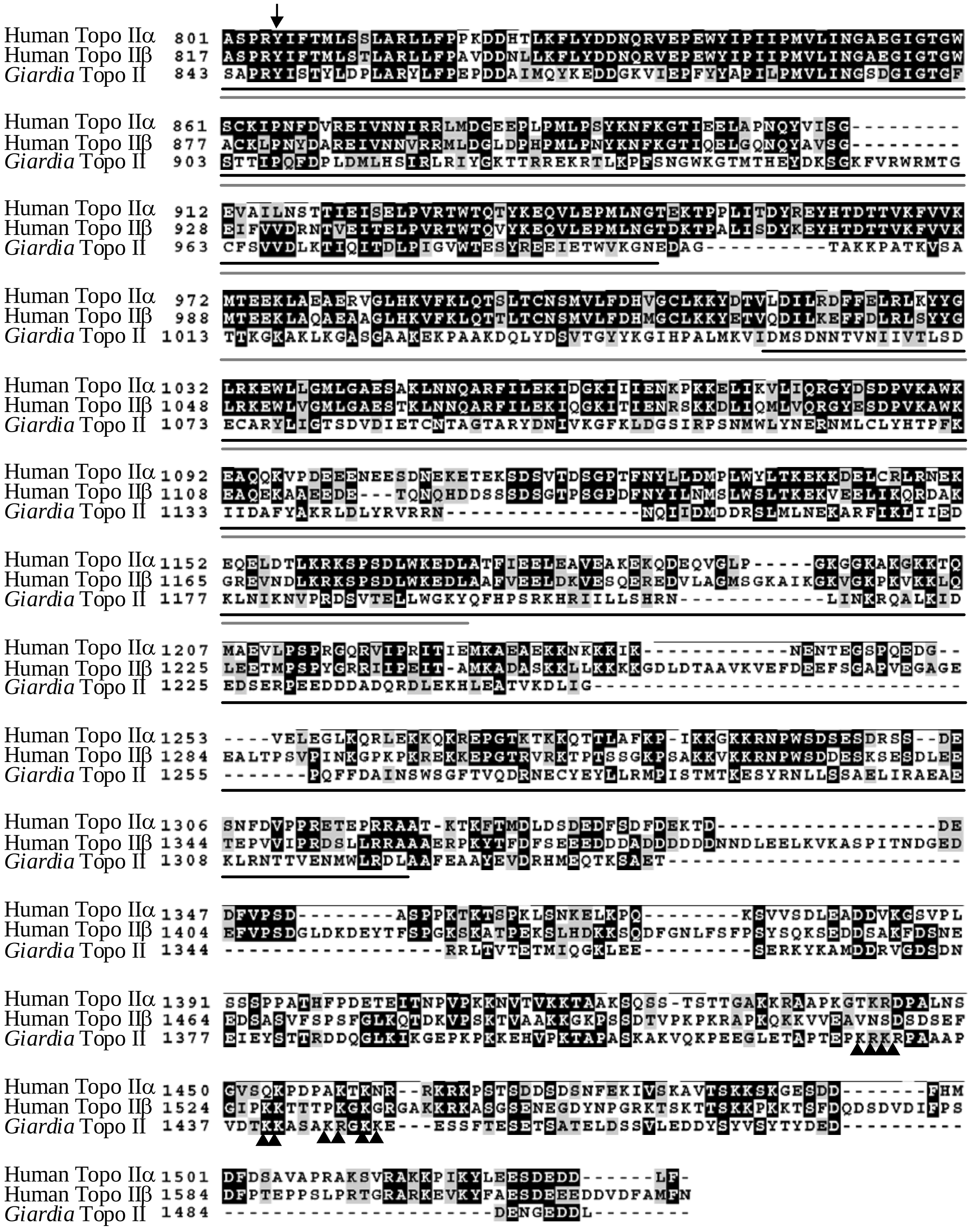

Fig. S1. Alignment of the full-length sequences of the Topo II proteins. The full-length sequences from the human topoisomerase IIα and topoisomerase IIβ, and *Giardia* Topo II (accession numbers are NP_001058.2, NP_001059.2, and XP_001708897.1, respectively) are analyzed by ClustalW 1.83

(http://www.clustal.org/). These Topo II proteins contain ATPase, gyrase B, and Topo IV domains, as predicted by pfam (http://pfam.sanger.ac.uk/)[65]. Letters in black boxes, letters in gray boxes and hyphens indicate identical amino acids, similar amino acids and gaps in the respective proteins, respectively. The conserved G-loop motif, GXXGXGXK [56], is indicated by asterisks. The catalytic important Tyr is pointed by an arrow. The conserved residues that interact with magnesium are indicated by circles. Two stretched of basic amino acids in the C terminal region are indicated by filled triangles. The conserved ATPase domains from these type II topoisomerases are indicated by dotted lines. The gyrase B domains from the human topoisomerase II$\alpha/\beta$ and *Giardia* Topo II proteins are indicated by gray and black boxes, respectively. The Topo IV domains from the human topoisomerase II$\alpha/\beta$ and *Giardia* Topo II are indicated by gray and black lines, respectively.
