# Supplementary material for: DNA Topoisomerase II Is Involved in Regulation of Cyst Wall Protein Genes and Differentiation in Giardia lamblia
Source: PLoS Negl Trop Dis. 2013 May 16;7(5):e2218. doi: 10.1371/journal.pntd.0002218 (PMC3656124; doi:10.1371/journal.pntd.0002218)
Supplement: Figure S2 — Alignment of the Topo IV domains of the Topo II proteins. (PDF) [file pntd.0002218.s002.pdf]

Figure S2

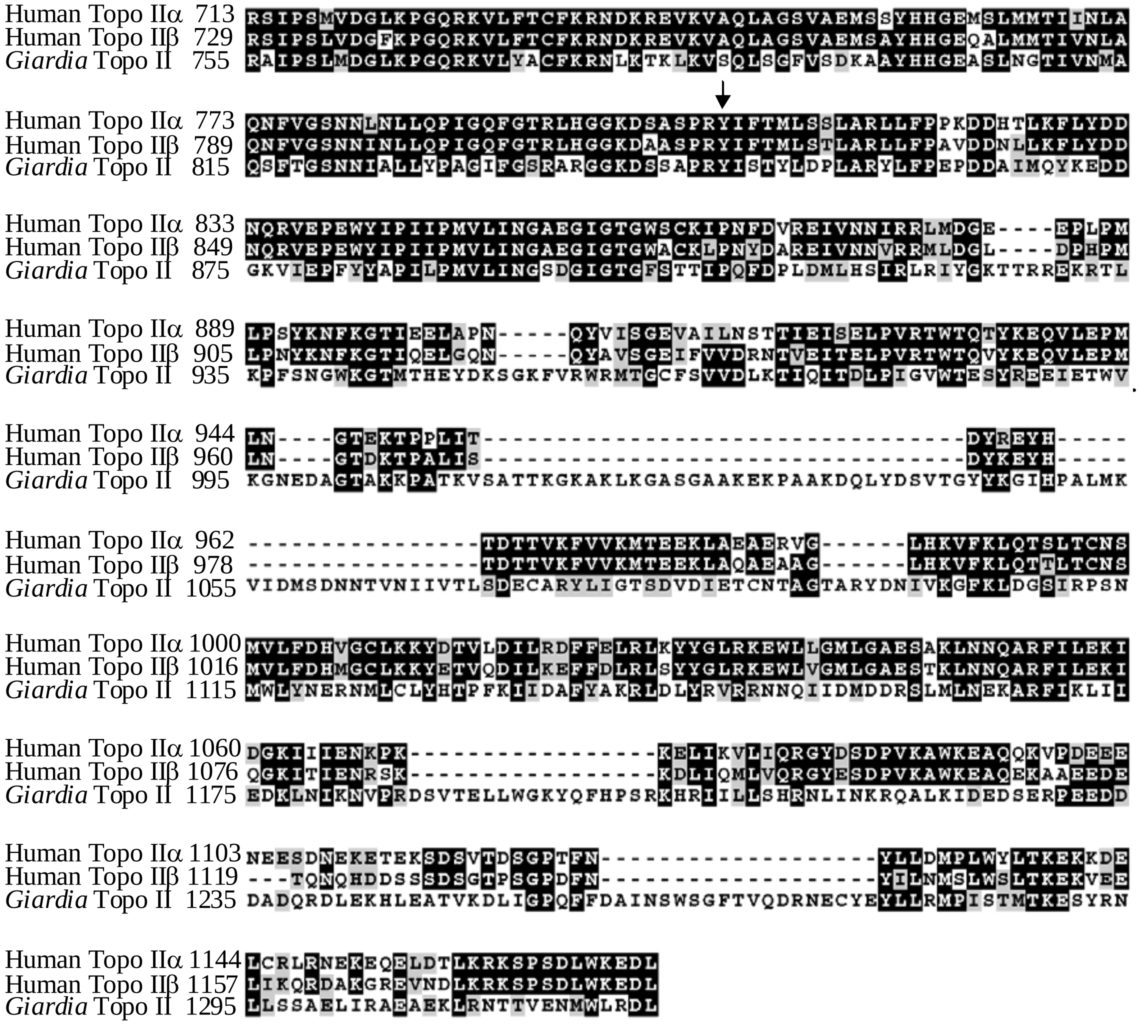

<u>Fig. S2.</u> Alignment of the Topo IV domains of the Topo II proteins. The Topo IV domains from the human topoisomerase IIα and topoisomerase IIβ, and *Giardia* Topo II are analyzed by ClustalW 1.83 (http://www.clustal.org/). These Topo IV domains were predicted by pfam (http://pfam.sanger.ac.uk/)[65]. Letters in black boxes, letters in gray boxes and hyphens indicate identical amino acids, similar amino acids and gaps in the respective proteins, respectively. The Tyr catalytic important Tyr is pointed by an arrow.
