# Supplementary material for: DNA Topoisomerase II Is Involved in Regulation of Cyst Wall Protein Genes and Differentiation in Giardia lamblia
Source: PLoS Negl Trop Dis. 2013 May 16;7(5):e2218. doi: 10.1371/journal.pntd.0002218 (PMC3656124; doi:10.1371/journal.pntd.0002218)
Supplement: Figure S3 — Phylogenetic analysis of Topo II proteins. (PDF) [file pntd.0002218.s003.pdf]

Figure S3

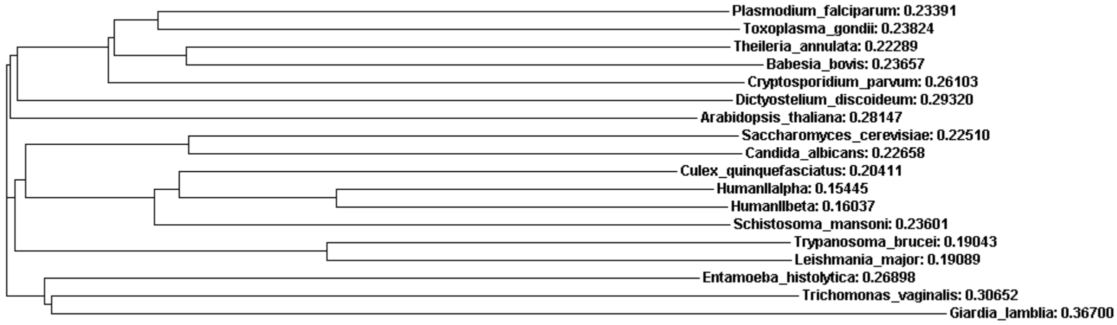

Fig. S3. Phylogenetic analysis of Topo II proteins. A neighbor-joining (Saitou, N. and Nei, M. (1987) Mol. Biol. Evol. 4, 406-425) phylogenetic tree was obtained from alignment of Topo II proteins from various organisms. GenBank accession numbers of the Topo II family are: Human IIalpha, (NP_001058.2), Human IIbeta (NP_001059.2), *Plasmodium falciparum* (XM_001348454.1), *Theileria annulata* (XP_952252.1), *Babesia bovis* (XP_001611613.1), *Toxoplasma gondii* (EEE25750.1), *Cryptosporidium parvum* (XP_625680.1), *Saccharomyces cerevisiae* (DAA10457.1), *Candida albicans* (XP_715530.1), *Arabidopsis thaliana* (NP_189031.1), *Dictyostelium discoideum* (XP_646786), *Leishmania major* (XP_003877071.1), *Culex quinquefasciatus* (EDS33447.1), *Entamoeba histolytica* (EAL46584.1), *Schistosoma mansoni* (XP_002577830.1), *Trichomonas vaginalis* (EAY12041.1), *Giardia lamblia* (EDO81223.1), and *Trypanosoma brucei* (XP_829219.1). The bootstrap values determined from 1000 trees are not shown. Values are higher than 400.
