# Supplementary material for: DNA Topoisomerase II Is Involved in Regulation of Cyst Wall Protein Genes and Differentiation in Giardia lamblia
Source: PLoS Negl Trop Dis. 2013 May 16;7(5):e2218. doi: 10.1371/journal.pntd.0002218 (PMC3656124; doi:10.1371/journal.pntd.0002218)
Supplement: Figure S4 — Induction of cwp1-3 and myb2 gene expression in the Topo II overexpressing cell line during encystation. (PDF) [file pntd.0002218.s004.pdf]

Figure S4

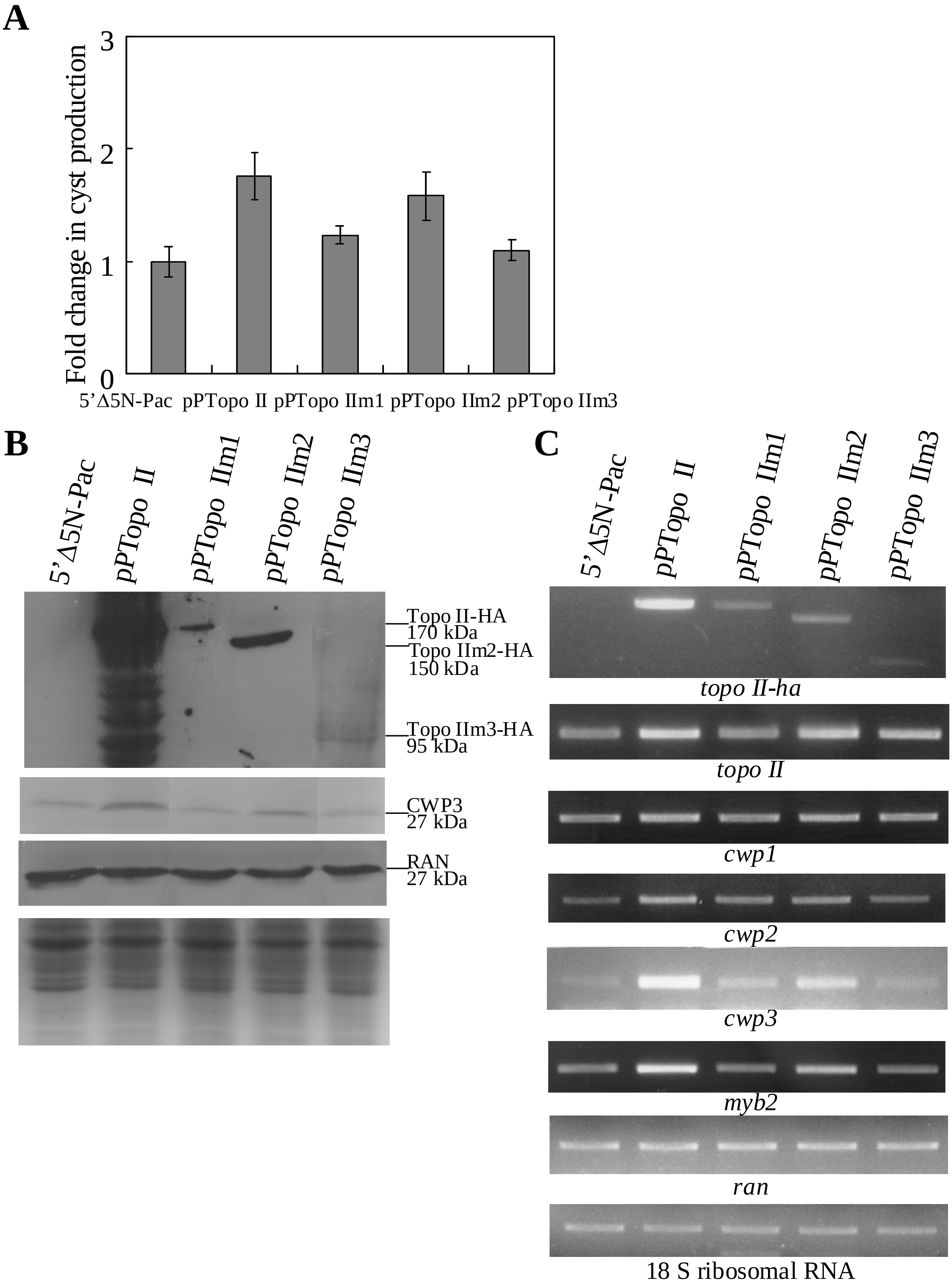

Fig. S4. Induction of *cwp1-3* and *myb2* gene expression in the Topo II overexpressing

cell line during encystation. (A) Cyst count. The 5’Δ5N-Pac, pPTopo II, pPTopo IIm1, pPTopo IIm2, and pPTopo IIm3 stable transfectants were cultured in encystation medium and then subjected to cyst count as described under “Experimental Procedures”. The sum of total cysts is expressed as relative expression level over control. Values are shown as means ± S. E. (B) Analysis of Topo II mutants. The 5’Δ5N-Pac, pPTopo II, pPTopo IIm1, pPTopo IIm2, and pPTopo IIm3 stable transfectants were cultured in encystation medium and then subjected to SDS-PAGE and Western blot. The blot was probed by anti-HA, anti-CWP3, and anti Ran antibodies. Equal amounts of protein loading were confirmed by SDS-PAGE and Coomassie Blue staining. Representative results are shown. (C) RT-PCR analysis of gene expression in the Topo II- and Topo II mutants- overexpressing cell lines. The 5’Δ5N-Pac, pPTopo II, pPTopo IIm1, pPTopo IIm2, and pPTopo IIm3 stable transfectants were cultured in encystation medium and then subjected to RT-PCR analysis. PCR was performed using primers specific for *topo II-ha*, *topo II*, *cwp1*, *cwp2*, *cwp3*, *myb2*, *ran*, and 18 S ribosomal RNA genes.
