# Supplementary material for: DNA Topoisomerase II Is Involved in Regulation of Cyst Wall Protein Genes and Differentiation in Giardia lamblia
Source: PLoS Negl Trop Dis. 2013 May 16;7(5):e2218. doi: 10.1371/journal.pntd.0002218 (PMC3656124; doi:10.1371/journal.pntd.0002218)
Supplement: Figure S5 — Analysis of Topo II function. (PDF) [file pntd.0002218.s005.pdf]

Figure S5

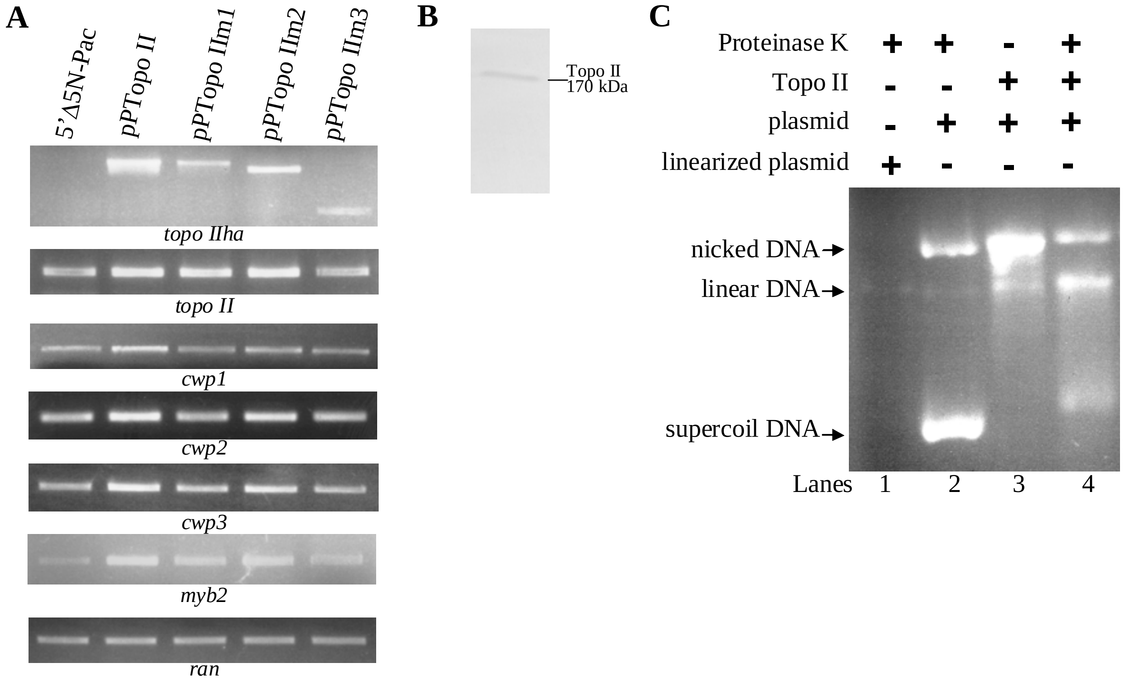

Fig. S5. Analysis of Topo II function. (A) RT-PCR analysis of gene expression in the Topo II- and Topo II mutants- overexpressing cell lines. The 5'Δ5N-Pac, pPTopo II, pPTopo IIm1, pPTopo IIm2, and pPTopo IIm3 stable transfectants were cultured in growth medium and then subjected to RT-PCR analysis again by using "Sense RNA sequence-specific priming" method (Michael C. Yeung, Allan S. Lau. Detection of Anti-Sense RNA Transcripts by Anti-Sense RT-PCR. RT-PCR Protocols. Methods in Molecular Biology Volume 193, 2002, pp 341-346.). For "Sense RNA sequence-specific priming" RT-PCR, 5 μg of DNase-treated total RNA was mixed with Superscript II RNase H- reverse transcriptase (Invitrogen) and primer mixtures including HAR, topo II1311R, cwp1R, cwp2R, cwp3R, myb2R, and ranR. Synthesized cDNA was used as a template in subsequent PCR. Semi-quantitative RT-PCR analysis of *topo II-ha*, *topo II*, *cwp1*, *cwp2*, *cwp3*, *myb2*, and *ran* gene expression was performed using primers topo IIHAF and HAR, topo II828F and topo II1311R, cwp1F and cwp1R, cwp2F and cwp2R, cwp3F and cwp3R, myb2F and myb2R, ranF and ranR, respectively. (B) Silver staining of purified Topo II. Recombinant Topo II protein was purified from *E. coli* using nickel affinity

chromatography under native conditions. Purified Topo II protein was analyzed by SDS-PAGE and silver staining. (C) DNA cleavage activity of Topo II with or without proteinase K. DNA cleavage assays were performed with purified recombinant Topo II and pUC119 plasmid (3.1kb). Components in the reaction are indicated above the lanes. Typically, 2 ng Topo II was mixed with 300 ng plasmid DNA. Some reaction mixtures was treated with proteinase K as a standard condition and one reaction mixture was stopped without proteinase K treatment (lane 3).
