# Supplementary material for: DNA Topoisomerase II Is Involved in Regulation of Cyst Wall Protein Genes and Differentiation in Giardia lamblia
Source: PLoS Negl Trop Dis. 2013 May 16;7(5):e2218. doi: 10.1371/journal.pntd.0002218 (PMC3656124; doi:10.1371/journal.pntd.0002218)
Supplement: Figure S6 — Binding of Topo IIC to vsp promoter. (PDF) [file pntd.0002218.s006.pdf]

Figure S6

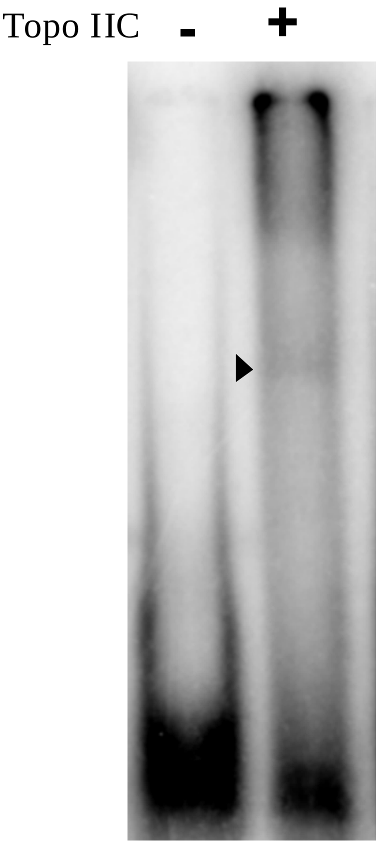

137620vsp-30/-1: GGGGCAGGGGCCACAGCAGGGGACCCTCTA

Fig. S6. Binding of Topo IIC to *vsp* promoter. Electrophoretic mobility shift assays were performed using purified Topo IIC and the 32P-end-labeled oligonucleotide probe 137620vsp-30/-1 (-30 to -1 relative to the translation start site of the *vsp* gene). Components in the binding reaction mixtures are indicated above the lanes. The arrowhead indicates the shifted complex.
