# Supplementary material for: DNA Topoisomerase II Is Involved in Regulation of Cyst Wall Protein Genes and Differentiation in Giardia lamblia
Source: PLoS Negl Trop Dis. 2013 May 16;7(5):e2218. doi: 10.1371/journal.pntd.0002218 (PMC3656124; doi:10.1371/journal.pntd.0002218)
Supplement: Table S1 — Oligonucleotides used in this study. (PDF) [file pntd.0002218.s007.pdf]

Table S1. Oligonucleotides used in this study.

Name	Sequence (5’ to 3’)
topo IIF	CACCATGGCCCAGAAGGCGAAG
topo IIR	AAGGTCATCTTCTCCATT
topo II828F	AATACCAATGACTCTGAC
topo II1311R	CTCCTCCGATGCCTTGGA
topo IIHAF	GATGGGTCGCACATTAAA
HAR	AGCGTAATCTGGAACATCGTATGGGTA
cwp1F	ATGATGCTCGCTCTCCTT
cwp1R	TCAAGGCGGGGTGAGGCA
cwp2F	ATGATCGCAGCCCTTGTTCTA
cwp2R	CCTTCTGCGGACAATAGGCTT
cwp3F	ATGTTTTCTCTGCTTCTTCT
cwp3R	TCTGTAGTAGGGCGGCTGTA
myb2F	ATGTTACCGGTACCTTCTCAGC
myb2R	GGGTAGCTTCTCACGGGGAAG
ranF	ATGTCTGACCCAATCAGC
ranR	GTTTACGGCCGGGAATACG
18SrealF	AAGACCGCCTCTGTCAATCAA
18SrealR	GTTTACGGCCGGGAATACG
topo IIrealF	CAAGGCGGCATACCATCAC
topo IIrealR	TCTGCGCCATGTTGACGAT
cwp1realF	AACGCTCTCACAGGCTCCAT
cwp1realR	AGGTGGAGCTCCTTGAGAAATTG
cwp2realF	TAGGCTGCTTCCCACTTTTGAG
cwp2realR	CGGGCCCGCAAGGT
cwp3realF	GCAAATTGGATGCCAAACAA
cwp3realR	GACTCCGATCCAGTCGCAGTA
myb2realF	TCCCTAATGACGCCAAACG
myb2realR	AGCACGCAGAGGCCAAGT
ranrealF	TCGTCCTCGTCGGAAACAA
ranrealR	AACTGTCTGGGTGCGGATCT
topo IIKF	TCGCCGGTACCTCTCCTACCTCATCATGTTCT
topo IIAR	GGCCGCCTAGGCTAAGCGTAATCTGGAACATCGTATGGGTAAAGGTCATCTT[illegible] TTTTC, underlined letters indicate the HA tag coding sequence

topo IIm1F	TGCTCCTCGTCACATTTCTACATA
topo IIm1R	TATGTAGAAATGTGACGAGGAGCA
topo IIm2AR	GGCCGCCTAGGCTAAGCGTAATCTGGAACATCGTATGGGTAGGTTTGCTCCAT[illegible] CGATC
topo IIm3AR	TCTTCCCTAGGCTAAGCGTAATCTGGAACATCGTATGGATACCTTGCAAGAGC[illegible] AAGTA
topo II5XF	GGCGTCTAGAGAGAGGCTATCCACACGGGGA
topo II5NR	GGCGCCATGGTTAATTTTTACATCGCAAACT
topo II5mNR	GGCGCCATGGTTAATTTTTACATCGCAAACTTCGGTGACCAAGCAAATTTCCT[illegible] CCAAGCTAGCAGGACATCGACGATAATA
topo IINR	GAGAAGCTGGCTTGTTTT
topo IICF	CACCATGGGCATCCCTAAGCTC
18S5F	CCAAAAAAGTGTGGTGCAGG
18S5R	GCCGGGCGCGGGCGCCGCGG
topo II5F	GTGCTTACTTGAAAGTAAAC
topo II5R	AATAGGTATAGGTTAGTGTGGG
cwp15F	CAACGGCTTACTAAATCATTCTCTTG
cwp15R	TTCTGTGTTTCTTGATCTGAGAGTTGT
cwp25F	CACTTTGATGAGAGCATGGG
cwp25R	TTAGTTCATATCTTAAGTTA
cwp35F	TGGGGGAGATAGGAGAATAC
cwp35R	ATCAGTAGTAACTTATTTTTTGGGAAAGAC
myb25F	TGGCTATGTATTTTTTCTTCTTCTACAGCT
myb25R	TAGCAGTACAGAGTAATTATTATTTTAGTA
ran5F	GTCCCAGCTGGGTTTGGCGAGATGCCTTGGA
ran5R	GCTACTCTCGGTTCCTGGGTTAAAGTTTTA
