# Supplementary material for: DNA Topoisomerase II Is Involved in Regulation of Cyst Wall Protein Genes and Differentiation in Giardia lamblia
Source: PLoS Negl Trop Dis. 2013 May 16;7(5):e2218. doi: 10.1371/journal.pntd.0002218 (PMC3656124; doi:10.1371/journal.pntd.0002218)
Supplement: Table S2 — Genes up or down regulated by Topo II overexpression (PDF) [file pntd.0002218.s008.pdf]

Table S2. Genes up or down regulated by Topo II overexpression

Number	Annotation	Orf number	Fold change (pPTopo II/5'Δ5N-Pac)*
1	Variant-specific surface protein (VSP)	137620	91.17
2	VSP	15206	48.99
3	VSP	105983	44.61
4	VSP	137610	44.46
5	VSP	41476	40.83
6	VSP	40630	32.07
7	VSP	13520	30.03
8	VSP	14307	24.79
9	VSP	102540	20.74
10	VSP	115085	19.40
11	VSP	13402	18.80
12	VSP	26590	17.69
13	VSP	137611	17.64
14	VSP	114122	14.57
15	High cysteine membrane protein group 1	25816	7.40
16	Hypothetical protein	105786	6.549
17	VSP	114277	6.47
18	VSP	112009	4.61
19	VSP	32933	4.29
20	VSP, putative	34442	4.23
21	VSP	114653	4.20
22	VSP	113093	3.95
23	High cysteine membrane protein group 4	114089	3.67
24	VSP	41539	3.62
25	VSP	112678	3.46
26	Protein 21.1	4846	3.41
27	Hypothetical protein	8960	3.39
28	Cyst wall protein 2	5435	3.15
29	VSP	113304	3.14
30	VSP	105759	2.99
31	Protein 21.1	15030	2.88

32	VSP	103992	2.84
33	VSP	103992	2.79
34	High cysteine membrane protein group 1	16318	2.78
35	VSP	103992	2.76
36	Hypothetical protein	13878	2.75
37	High cysteine membrane protein group 1	11309	2.71
38	VSP	111873	2.70
39	VSP	111874	2.67
40	High cysteine membrane protein group 1	10659	2.64
41	VSP	112867	2.64
42	VSP	114162	2.60
43	Hypothetical protein	16078	2.57
44	VSP	114121	2.54
45	VSP, putative	103916	2.50
46	Hypothetical protein	114210	2.45
47	VSP	8338	2.44
48	VSP	112048	2.42
49	High cysteine membrane protein group 3	114891	2.42
50	High cysteine membrane protein group 1	7715	2.39
51	Cyst wall protein 1	5638	2.39
52	VSP	118900	2.37
53	VSP	113163	2.36
54	Variant-specific surface protein VSP4A1 precursor	101498	2.34
55	Hypothetical protein	8654	2.31
56	High cysteine membrane protein group 1	91707	2.3
57	VSP	97233	2.29
58	TM efflux prot	14247	2.29
59	High cysteine membrane protein group 6	113512	2.28
60	DNA topoisomerase II	16975	2.27

61	VSP	41472	2.25
62	Cathepsin B precursor	16779	2.24
63	VSP presumed INR	137714	2.24
64	Hypothetical protein	23934	2.23
65	Hypothetical protein	89815	2.22
66	Hypothetical protein	39305	2.20
67	Hypothetical protein	30764	2.19
68	High cysteine membrane protein group 1	32607	2.19
69	Variant-specific surface protein	9276	2.18
70	VSP	137708	2.17
71	Hypothetical protein	18722	2.17
72	VSP	114065	2.17
73	Hypothetical protein	32657	2.16
74	VSP with INR	11470	2.15
75	VSP	112113	2.13
76	Hypothetical protein	5206	2.12
77	Hypothetical protein	36883	2.11
78	VSP with INR	115797	2.10
79	High cysteine membrane protein group 6	114470	2.10
70	Hypothetical protein	28112	2.09
81	Hypothetical protein	117441	2.08
82	VSP	115742	2.08
83	High cysteine membrane protein EGF-like	114991	2.07
84	High cysteine protein	137732	2.06
85	Hypothetical protein	116865	2.05
86	Sugar transport family protein	9046	2.05
87	VSP	11521	2.04
88	VSP	115796	2.04
89	VSP	101410	2.04
90	VSP	135919	2.04
91	High cysteine membrane protein EGF-like	16936	2.04

92	CEGP1 protein	17120	2.02
93	Amino acid transporter family	16283	2.02
94	VSP	32916	2.00
95	VSP	135882	2.00
96	VSP	137612	0.09
97	VSP with INR	119707	0.13
98	VSP	90215	0.14
99	VSP	34357	0.14
100	VSP	40571	0.14
101	VSP with INR	16501	0.17
102	VSP	115047	0.20
103	VSP	137617	0.20
104	VSP	114672	0.36
105	Hypothetical protein	99726	0.37
106	VSP	40591	0.37
107	Hypothetical protein	92625	0.37
108	VSP AS8	13194	0.38
109	Protein 21.1	17562	0.42
110	Hypothetical protein	14117	0.47
111	VSP	137697	0.48
112	Zinc finger protein, putative	15295	0.49
113	Hypothetical protein	10238	0.49
114	Glucosamine-6-phosphate isomerase, putative	15276	0.49
115	VSP S8	137604	0.50

**p* values were determined to <0.05 for groups in which the average means changed by a factor of ≥2.0 or ≤0.5.
