# Supplementary material for: DNA Topoisomerase II Is Involved in Regulation of Cyst Wall Protein Genes and Differentiation in Giardia lamblia
Source: PLoS Negl Trop Dis. 2013 May 16;7(5):e2218. doi: 10.1371/journal.pntd.0002218 (PMC3656124; doi:10.1371/journal.pntd.0002218)
Supplement: Table S3 — Genes up or down regulated by etoposide treatment (PDF) [file pntd.0002218.s009.pdf]

Table S3. Genes up or down regulated by etoposide treatment

Number	Annotation	Orf number	Fold change (+etoposide/-etoposide)*
1	High cysteine membrane protein group 1	25816	4.47
2	Variant-specific surface protein (VSP)	112113	3.58
3	Hypothetical protein	99213	3.38
4	VSP	9276	3.05
5	VSP	136004	2.96
6	VSP	14331	2.93
7	VSP	41626	2.81
8	VSP	137744	2.79
9	Hypothetical protein	16135	2.62
10	Hypothetical protein	87955	2.61
11	Hypothetical protein	137713	2.49
12	VSP	41476	2.45
13	VSP	101380	2.45
14	VSP	115047	2.37
15	Hypothetical protein	34334	2.35
16	DNA repair protein RAD51	13104	2.35
17	Ribosomal protein S27	10780	2.34
18	VSP with INR	112208	2.32
19	VSP	38901	2.31
20	High cysteine protein	101589	2.30
21	VSP with INR	137613	2.29
22	Hypothetical protein	10763	2.28
23	VSP with INR	115797	2.27
24	Hypothetical protein	27652	2.24
25	Hypothetical protein	91187	2.23
26	VSP	15400	2.21
27	Kinesin-3	112846	2.21
28	VSP, putative	103142	2.21
29	VSP	137691	2.20
30	Hypothetical protein	7610	2.18
31	VSP	115796	2.18

32	VSP	137729	2.17
33	VSP	137723	2.15
34	VSP	41227	2.13
35	Multidrug resistance-associated protein 1	41118	2.13
36	VSP with INR	101010	2.13
37	Kinase, CMGC CDK	11290	2.11
38	Flavohemoprotein lateral transfer candidate	15009	2.10
39	Hypothetical protein	112696	2.10
40	Hypothetical protein	8394	2.08
41	Multidrug resistance-associated protein 1	115052	2.08
42	VSP	103992	2.08
43	Hypothetical protein	9355	2.07
44	Hypothetical protein	95451	2.05
45	Hypothetical protein	27767	2.05
46	Hypothetical protein	8377	2.05
47	VSP	111936	2.04
48	Hypothetical protein	7232	2.04
49	VSP	113357	2.03
50	Transcription factor RRN3	11742	2.02
51	VSP	16472	2.02
52	Dynein heavy chain	16804	2.02
53	AAA family ATPase	16867	2.01
54	Tenascin precursor	114815	2.01
55	Hypothetical protein	10861	2.01
56	Hypothetical protein	31420	2.00
57	Hypothetical protein	16070	0.31
58	Hydroxymethylglutaryl-CoA synthase	13962	0.32
59	Hypothetical protein	17468	0.33
60	Hypothetical protein	2267	0.33
61	Hypothetical protein	4852	0.34

62	Protein 21.1	93011	0.34
63	Hypothetical protein	17375	0.36
64	Hypothetical protein	6185	0.36
65	Hypothetical protein	10014	0.39
66	Hypothetical protein	14401	0.39
67	Kinase, NEK	16122	0.40
68	Hypothetical protein	6471	0.40
69	Microsomal signal peptidase 18 kDa subunit	9174	0.41
70	Protein 21.1	23492	0.41
71	Deoxyguanosine kinase/deoxyadenosine kinase subunit, putative	4558	0.42
72	Hypothetical protein	16844	0.42
73	Hypothetical protein	3720	0.42
74	Hypothetical protein	6026	0.43
75	Hypothetical protein	12229	0.44
76	Serine/threonine protein Kinase	5811	0.44
77	Hypothetical protein	6245	0.44
78	Hypothetical protein	15419	0.45
79	Protein 21.1	16532	0.45
80	SALP-1	4410	0.46
81	Hypothetical protein	33721	0.46
82	Hypothetical protein	12225	0.47
83	Axoneme-associated protein GASP-180	13475	0.47
84	H-SHIPPO 1	103164	0.47
85	Hypothetical protein	39159	0.47
86	Hypothetical protein	41451	0.48
87	Hypothetical protein	15290	0.48
88	Hypothetical protein	4690	0.48
89	Hypothetical protein	4692	0.48
90	RAD50 DNA repair protein, putative	17495	0.49
91	Hypothetical protein	16581	0.49
92	Histone H3	135231	0.49

93	Protein 21.1	17046	0.49
94	Molybdopterin biosynthesis MoeB protein	11436	0.49
95	Impact-like protein	15984	0.49
96	Hypothetical protein	29179	0.49
97	Hypothetical protein	8250	0.49
98	Hypothetical protein	5615	0.49
99	Hypothetical protein	96818	0.50
100	Hypothetical protein	9717	0.50
101	Kinase, NEK	10893	0.50
102	Hypothetical protein	15499	0.50
103	Protein 21.1	101699	0.50
104	Hypothetical protein	10696	0.50

*p values were determined to be <0.05 for groups in which the average means changed by a factor of ≥ 2.0 or ≤ 0.5.
